# Unraveling sex differences in age-related hippocampal decline: differential mitochondrial dysfunction, Lonp1-dependent mitochondrial proteostasis and mtROS production in aged C57BL/6 mice

**DOI:** 10.1038/s41419-025-08360-y

**Published:** 2025-12-30

**Authors:** Karina A. Cicali, Claudia Jara, Daniela Cortés-Díaz, Matías Lira, Ítalo Fuentes, Alejandra Catenaccio, Josefa Arnaíz, Micaela Ricca, Sebastián Valenzuela, Carolina A. Oliva, Daniela S. Rivera, Cheril Tapia-Rojas

**Affiliations:** 1 Laboratory of Neurobiology of Aging, Centro Científico y Tecnológico de Excelencia Ciencia & Vida, Fundación Ciencia & Vida, Huechuraba, Santiago Chile; 2 Facultad de Ciencias, Universidad San Sebastián, Lota 2465, Santiago, Chile; 3 Centro Científico y Tecnológico de Excelencia Ciencia & Vida, Fundación Ciencia & Vida, Avenida del Valle Norte 725, Huechuraba, Santiago Chile; 4 Facultad de Medicina Veterinaria, Universidad San Sebastián, Bellavista 07, Recoleta, Santiago, Chile; 5 Centro para la Transversalización de Género en I + D +i + e, Vicerrectoría de Investigación y Doctorados, Universidad Autónoma de Chile, Santiago, Chile; 6 GEMA Center for Genomics, Ecology and Environment, Facultad de Ciencias, Ingeniería y Tecnología, Universidad Mayor, Santiago, Chile

**Keywords:** Cognitive ageing, Protein quality control

## Abstract

Aging is a progressive process characterized by cellular and molecular damage leading to mitochondrial dysfunction and cognitive decline. Mitochondrial dysfunction is a critical factor in memory impairment in aging and neurodegenerative diseases. While sex differences in aging have been observed across various species, the underlying cellular and molecular mechanisms remain poorly understood, mainly focused on mitochondrial proteostasis. This study examined hippocampal-dependent cognitive decline and mitochondrial dysfunction in aged male and female C57BL/6 J mice. Our results reveal sex-dependent differences in cognitive impairment, with aged males exhibiting more significant deficits in spatial and localization memory, while aged females show impairments in recognition memory. Additionally, aged males display increased oxidative stress and exacerbated mitochondrial superoxide production, leading to more severe bioenergetic deficiencies. Conversely, aged females exhibit heightened mitochondrial permeability transition pore (mPTP) activity, suggesting a distinct mechanism of mitochondrial dysfunction, which could explain, almost in part, the cognitive differences in aging. Investigating possible mechanisms responsible for this mitochondrial dysfunction, we found that mitochondrial proteostasis is more prone to failure in aged males, with a significant decrease in the protease activity of Lonp1, a key matrix mitochondrial protease degrading >50% of the mitochondrial proteome. To further reinforce these findings, we replicated key experiments in SAMP8 mice, a model of accelerated aging, obtaining consistent results that strengthen the robustness and generalization of our conclusions. These findings suggest that sex influences hippocampal aging at multiple levels, highlighting the need to consider sexual dimorphism in aging research. This study also emphasizes the critical role of mitochondrial proteostasis in maintaining mitochondrial function in aging in a sex-dependent manner. Understanding these differences could facilitate the development of sex-specific strategies to mitigate age-related cognitive decline and neurodegeneration.

## Introduction

Aging is a natural process characterized by the gradual decline of physical and cognitive capacities due to the accumulation of molecular and cellular damage [1]. In recent decades, there has been an increase in the aged world population, with women exhibiting a longer life expectancy compared to men [2, 3]. Among 90-year-olds, there are approximately 50 men for every 100 women; whereas among 100-year-olds, the ratio decreases to about 25 men for every 100 women [4]. This disparity in survival is also observed in non-human mammals, where, despite similar aging rates between the sexes, a clear trend toward longer life expectancy is observed in females [5]. Although it is suggested that these differences may be due to biological and sociocultural factors [4], the cellular and molecular bases that lead females to live longer than males are still largely unknown, and a better understanding of the mechanisms underlying sex differences in aging is of great relevance. More relevant is that despite females having more extended lives, they are more prone to suffer neurodegenerative diseases, such as Alzheimer’s Disease (AD), for unclear reasons [6, 7]. The brain is one of the organs most affected by aging [8], presenting impaired neuroplasticity, abnormal neuronal network activity, decreased bioenergetics, dysregulation of neuronal calcium homeostasis, protein accumulation, and increased oxidative damage and inflammation [9]. In particular, the hippocampus, a brain structure recognized for its roles in learning and memory [10], undergoes morphological changes and a progressive decrease in both synaptic plasticity and function throughout the aging process [11–13]. Sex differences have been observed in hippocampal morphology, plasticity, cognition, and susceptibility to disorders that affect its structure and function. Females have larger primary dendrites and more branching points in the basal dendrites of pyramidal neurons in the CA3 than males, as well as more significant cell proliferation in the dentate gyrus and CA1 [14]. These differences also manifest in the susceptibility to stress-induced cognitive disorders, where males exhibit worse performance in spatial memory than females [14, 15].

Among the mechanisms that contribute to the decrease in hippocampal function during aging is the increase in oxidative stress [9], which is caused by the rise in the production of reactive oxygen and nitrogen species (ROS/RNS) and a reduction in the antioxidant defenses [16]. Sex-dependent differences in oxidative stress have been reported in adult animals, with males exhibiting higher levels than females [17, 18]. For example, the levels of the enzyme NADPH oxidase (NOX), which is a key generator of oxidative stress in cells [19], are higher in males compared to females [20–22]. Despite this evidence, a critical gap remains in understanding how sex differences in oxidative stress manifest in the aging hippocampus and contribute to cognitive impairment, underscoring the importance of investigating other critical contributors to ROS generation. In this context, the mitochondria and their potential sex-specific regulation promoting oxidative damage during aging remain unexplored.

Mitochondria are one of the major producers of ROS in cells. This organelle is essential for generating energy as ATP through oxidative phosphorylation (OXPHOS), maintaining oxidative balance, and regulating calcium homeostasis [23, 24]. In addition, mitochondria present their own machinery that allows them to maintain the homeostasis of the mitochondrial proteome [25], including chaperones and proteases, such as Lonp1, known as the guardian of the mitochondrial proteome [26, 27]. During the aging process, the structure and functionality of mitochondria deteriorate due to impaired mitochondrial quality control mechanisms and increased oxidative damage [28–30]. This age-related mitochondrial dysfunction leads to cognitive decline in aged animals [13]. Like other cellular proteins, mitochondrial proteins unfold, misfold, and oxidize due to increased ROS and aggregate over time [25]. Specifically, damaged OXPHOS proteins are found to disrupt the overall structure and function of mitochondria, particularly in aged organisms [31]. Excessive ROS also contributes to the aging phenotype and the development of neurodegenerative diseases. Nevertheless, how these alterations in mitochondrial function and proteins crucial to maintaining mitochondrial proteostasis might manifest differentially between the sexes in aging has not been studied in depth.

Here, we analyzed cognitive differences between aged male and female C57BL/6 J mice and their association with mitochondrial dysfunction. Our findings reveal that aged males experience delayed spatial learning and impaired short-term memory, which correlate with significantly elevated mitochondrial superoxide production and increased oxidative stress within the hippocampus. Additionally, mitochondrial protein quality control appears to be more compromised in aged males, as evidenced by reduced Lonp1 protease activity and increased accumulation of unfolded proteins. In contrast, aged females exhibit impairments in recognition memory and increased mitochondrial permeability transition pore (mPTP) activity, indicating a greater susceptibility to mitochondrial permeability changes and impaired calcium homeostasis. These results indicate that the aging process affects males and females differently, with aged males exhibiting higher oxidative stress and proteostasis failure. In contrast, aged females exhibit increased mitochondrial calcium vulnerability, which may contribute to sex-specific differences in aging and life expectancy.

## Materials and methods

### Animals

Adult and aged C57BL/6 J and SAMP8 mice (Senescence-Accelerated Mouse Prone 8) were obtained from Fundación Ciencia and Vida. These animals were housed and maintained at 24 °C on a 12:12 h light-dark cycle, with food and water provided ad libitum. Male and female 3 and 20-month-old C57BL/6 J mice, and 2 and 10-month-old SAMP8 mice were used. In our study, the total sample consisted of ≥10 animals per sex and age class (adult male, adult female, aged male, aged female; *n* = 40). We utilized all animals for the behavioral assays, while for brain biochemistry, mitochondrial function, and immunohistochemical assays, we analyzed ≥5 animals per group, increasing to ≥ 6 in cases where preliminary trends suggested more significant differences. The statistical framework for determining sample size and detectable effects was based on standard power analysis methods, using G*Power-equivalent settings [32, 33]. We conducted a sensitivity analysis with α = 0.05 and power (1–β) = 0.80 to estimate the smallest effect sizes detectable with our biochemical sample sizes.

#### Main effects

For a 2 × 2 design, considering a main effect with two levels (e.g., females vs. males) and collapsing across the other factor (age), the analysis reduces to a two-sample t-test: *n* = 5 per condition → 10 subjects per factor level; and *n* = 6 per condition → 12 subjects per factor level. Using G*Power, we calculated the minimum detectable effect sizes: With *n* = 5 per condition, detectable Cohen’s d ≈ 1.33, corresponding to Cohen’s f ≈ 0.66 and partial η² ≈ 0.31. With *n* = 6 per condition, detectable Cohen’s d ≈ 1.20, f ≈ 0.60, and partial η² ≈ 0.26. Thus, for main effects, our study is powered to detect large differences, where the factor explains approximately 26–31% of the variance.

#### Interaction effects

For the Sex × Age interaction in the 2 × 2 ANOVA (4 cells total): *n* = 5 per condition (*N* = 20 total) → detectable Cohen’s f ≈ 0.84, partial η² ≈ 0.41; and for *n* = 6 per condition (*N* = 24 total) → detectable f ≈ 0.74, η² ≈ 0.36. These values indicate that the design can detect only very large interactions, explaining approximately 36–41% of the variance. Therefore, with 5–6 animals per group, our study is well-powered to detect large, meaningful differences between sexes, ages, or their interaction, such as pronounced shifts in biochemicals. However, smaller or more subtle effects may go undetected, and we acknowledge this as a limitation of this study.

The animals were handled according to the National Institute of Health guidelines (NIH, Baltimore, MD, USA). The experimental procedures were approved by the Bioethical and Biosafety Committee of Fundación Ciencia y Vida. This study was conducted in accordance with the ARRIVE guidelines.

### Hormone levels

To determine the changes in sexual hormones in aging, we measured the levels of these hormones in adult and aged females and testosterone in adult and aged males. Blood samples were collected in the presence of an anticoagulant (*n* = 5 per group). Serum estradiol levels were quantified in females and testosterone levels in males using specific assays. The results are shown in the following table:

**Table:** 

Testosterone (ng/ml)		Estradiol (pg/ml)	
Adult	Old	Adult	Old
0,35	0,97	6,83	7,2
3,01	0,1	4,72	6,52
0,07	0,99	2,9	4,25
0,362	0,1	9,8	4,19
0,15	0,25	6,15	5,92

To account for the high variability typical of hormone measurements, we analyzed age-related differences using geometric means (GM) derived from log10-transformed values with 95% confidence intervals (CI). Comparisons between age groups (Adult vs. Old) were performed using Welch’s t-test. The fold-change was calculated as the ratio of the geometric means. The use of geometric means is particularly appropriate for skewed biological data, as it reduces the influence of extreme values and provides a more robust measure of central tendency.

### Behavioral tests

All behavioral tests were monitored by Any-MAZE Behavioral software (Stoelting Co), using the chambers and instruments manufactured or recommended by the manufacturer. All behavioral tests were performed in the 12 h light phase of the animal’s light/dark cycle.

#### Open field test

The animals were individually placed at the center of a 72 × 72 x 32 cm white acrylic box and were allowed to move freely for 10 min as previously described [34, 35]. The distance traveled, speed, and number of entrances to the center of the box were recorded.

#### Morris water maze (MWM) test

The MWM test was used as a spatial memory behavioral test as previously described [13, 35, 36]. Mice were trained in a 1.2 m diameter circular pool (opaque water, 50 cm deep) filled with 19–21 °C water. A submerged 9 cm platform (1 cm below the surface, invisible to the animal) was used for training, with a maximum trial duration of 60 s and 2 s on the platform at the end of the trials. Each animal was trained to locate the platform. The test was performed with three trials per day for 10 days except for weekends, and a probe test was carried out on day 11, when the platform was removed. The latency time (in seconds) required for the animal to reach the platform and the time spent in each quadrant (in seconds) was measured. After testing, the mouse was gently removed from the maze and returned to its cage. Spatial acuity was calculated using the time mice spent in the quadrant after the platform was removed on the probe day and the time mice spent in the platform area. Both times were multiplicated to obtain the spatial acuity score. Data was plotted as the sum of escape latency (from the 10 days of learning phase) in the X axis, versus the spatial acuity score in the Y axis.

#### Novel object recognition (NOR) and novel object localization (NOL) tests

NOR and NOL tests were performed in a 38 × 38 × 32 cm acrylic box as previously described [13]. The animals were habituated in the box for 5 minutes twice on the first day without any object. The next day, for the NOR test, each animal was placed in the box containing two identical objects (old objects) for 10 min. After 2 h, the animal was exposed to one of the old objects and a new object of different shape and color. The recognition index was calculated as the time spent by the mouse exploring the new object divided by the time spent exploring both objects. The box and objects were cleaned (50% ethanol) between each animal testing. NOL test was performed 2 h after NOR test; the animals were exposed to the same old object, and the previous novel object’s location was changed. The localization recognition index was calculated by dividing the time the animals spent exploring the new object by the time exploring both objects. After each test, the box chamber was cleaned with ethanol before a different mouse was tested.

### Reagents and antibodies

The primary antibodies used were, rabbit anti-GAPDH (1:1000, sc-25778, Santa Cruz Biotechnology, Inc. USA), mouse anti-4-HNE (H6275-02, US Biological, Life Sciences, 1:1,000), mouse anti-catalase (1:1000, sc- 271803, Santa Cruz Biotechnology, Inc. USA), mouse anti-SOD1 (1:1000, sc-271014, Santa Cruz Biotechnology, Inc. USA), mouse anti-Glutathione reductase (1:1000, sc-133245, Santa Cruz Biotechnology, Inc. USA), mouse anti-OSCP (1:1000, sc-365162, Santa Cruz Biotechnology, Inc. USA), mouse anti-Cyp-D (1:1000, sc-37606, Santa Cruz Biotechnology, Inc. USA), mouse anti-ANT (1:1000, sc-293434, Santa Cruz Biotechnology, Inc. USA), mouse anti-VDAC (1:100, sc-390996; Santa Cruz Biotechnology, Inc. USA) mouse anti-Total OXPHOS Human WB Antibody Cocktail (1:1000, ab110411, Abcam, Inc.), mouse anti-mtHsp70 (1:1000, MA3-028, Thermo Fisher Scientific, USA), mouse anti-Hsp60 (1:1000, MA3-012, Thermo Fisher Scientific, USA), mouse anti-Oma1 (1:1000, sc-515788, Santa Cruz Biotechnology, Inc. USA), mouse anti-ClpP (1:1000, sc-271284, Santa Cruz Biotechnology, Inc. USA), rabbit anti-Lonp1 (1:1000, PA5-51692, Thermo Fisher Scientific, USA). The fluorescent dyes used were MitoTracker Red CM-H2Xros (Catalog number: M7513, Thermo Fisher Scientific, USA) and CM-H2DCFDA (Catalog number: C6827, Thermo Fisher Scientific, USA).

### Immunoblotting

The hippocampus of the 3- and 20-month-old male and female mice were dissected on ice and immediately processed as previously described [28, 37]. The hippocampal tissue was homogenized in HEPES buffer (25 mM Hepes, 125 mM NaCl, 25 mM NaF, 1 mM EDTA, 1 mM EGTA, 1% NP-40, pH = 7,4), supplemented with a protease inhibitor mixture (catalog number 78429, Thermo Fisher Scientific) and phosphatase inhibitors (NaF 25 mM, Na_2_P_2_O_7_ 30 µM, Na_3_VO_4_ 100 mM) using a homogenizer and then sequentially passed through pipette tips of different volumes. The protein samples were centrifuged at 14,000 rpm for 20 min at 4 °C. The protein concentrations were determined using the BCA Protein Assay Kit (Catalog number 23225, Pierce, Rockford, IL, USA). Samples were resolved by SDS-PAGE, followed by immunoblotting on PVDF membranes. The membranes were incubated with the primary antibodies and anti-mouse or anti-rabbit IgG peroxidase-conjugated antibodies (Pierce) and visualized using an ECL kit (Luminata Forte Western HRP substrate, Millipore, USA).

### Obtaining hippocampal slices

Animals were anesthetized and then perfused through the heart with 1x PBS, followed by fixation with 4% paraformaldehyde (PFA, 4896610S, Merck) in 0.1 M phosphate buffer for 30 min. Brains were postfixed overnight at 4 °C in 4% PFA and washed 10 min, 3 times in 1x PBS. A sucrose gradient (10% and 20%, 2 h each, at room temperature and 30% overnight at 4 °C) was performed. Then, the whole brain was immersed in OCT Compound (4583, Sakura). Coronal sections 25 μm thick were collected from anterior to posterior on a Leica cryostat in a 24-well plate with 1x PBS and kept at 4 °C until use. Representative sections from each animal were mounted on Premium Micro Slide (PC2-302-16, PR PorLab) in 1x PBS, allowed to dry at room temperature, and stored at -20 °C until use [38, 39].

### Immunofluorescence (IF)

Slides were removed from -20 °C and left at room temperature; then, IF was performed. Briefly, they were hydrated in 1x PBS and incubated in blocking/permeabilization solution (Triton X-100 0.5%, BSA 5% in 1x PBS) for 1 h at room temperature. Primary antibodies were diluted in this solution and incubated at 4 °C overnight. After 3 washes in 1x PBS, 10 min each, the secondary antibodies were diluted in the same solution, incubated for 2 h at room temperature, and washed again 10 min, 3 times in 1x PBS. The slides were coated with Fluoromount-G (00-4958-02, Invitrogen, USA). Antibodies used were: mouse anti-4HNE (1:300; 298112, USBiological), mouse anti Lonp1 (1:500; sc-293244, Santa Cruz Biotechnology, Inc. USA) and anti-mouse Alexa 488 (1:500; A-21202, Invitrogen, USA) [37, 38].

### Isolation of an enriched-mitochondrial fraction from the hippocampus

As previously described, a fraction enriched in mitochondria was isolated from the hippocampus [28, 37]. Hippocampal tissue was homogenized in MSH buffer (230 mM mannitol, 70 mM sucrose, 5 mM Hepes, pH 7.4), supplemented with a protease and phosphatase inhibitor cocktail in a glass homogenizer. Homogenates were centrifuged at 600 × *g* for 10 min at 4 °C. The supernatant was centrifuged at 8000 × *g* for 10 min; the newly enriched mitochondrial pellet was resuspended in respiration buffer or HEPES buffer whenever stated. Both cytoplasmic and mitochondrial fractions were obtained. Protein concentration was determined by using a standard BCA kit (Thermo Fisher Scientific, USA).

### Measurement of ATP and mitochondrial membrane potential (MMP)

ATP concentration was measured in tissue lysates obtained with HEPES buffer (25 mM HEPES, 125 mM NaCl, 25 mM NaF, 1 mM EDTA, 1 mM EGTA, 1% NP-40, pH = 7,4) using a luciferin/luciferase bioluminescence assay kit (ATP determination kit no. A22066, Molecular Probes, Invitrogen, USA) [28, 37]. MMP was measured in a mitochondrial-enriched fraction (50 μg) diluted in 100 μL of KCl respiration buffer and incubated at 37 °C for 30 min with MitoTracker Red CM-H2Xros [28]. Samples were centrifuged, and the fluorescence was measured at 590 nm in the resuspended mitochondrial pellet. The ATP production was measured in the 25 μg mitochondrial-enriched fraction supernatant after 30 min incubation with the oxidative substrates pyruvate and malate using a luciferin/luciferase bioluminescence assay kit.

### Measurement of ROS content and superoxide production

ROS content was measured using the fluorescent dye CM-H2DCFDA (DCF) (Thermo Fisher Scientific, USA). 25 ug of hippocampal protein samples were incubated with 25 μM of DCF. Fluorescence was measured in BioTek Synergy HT (485 nm, 530 nm) [37]. The fluorescent dye MitoSox (Thermo Fisher Scientific, USA) measured the superoxide production as described previously [28, 37]. Briefly, a fraction enriched in mitochondria (25 μg of protein) was isolated from the hippocampus and diluted in 100 μL of KCl respiration buffer. It was incubated with pyruvate and malate as oxidative substrates and the fluorescent dye MitoSox. Then, mitochondria were incubated at 37 °C for 30 min and centrifuged at 8000 × *g* for 10 min at 4 °C. After this time, mitochondria were resuspended and superoxide production was measured in BioTek Synergy HT (530 nm, 590 nm).

### Mitochondrial permeability transition pore (mPTP) assay

Mitochondrial permeability transition pore (mPTP) activity was measured in a mitochondrial-enriched fraction (50 μg of protein) [28], which were loaded with the acetoxymethyl ester of calcein dye, calcein AM, that passively diffuses into the cells and accumulates in cytosolic compartments, including the mitochondria. In addition, CoCl_2_ was added. The cobalt quenches the fluorescence from cytosolic calcein; in contrast, in a condition of closed mPTP, mitochondrial fluorescence is maintained. However, when the mPTP is open, green mitochondrial calcein fluorescence loss occurs.

### Unfolded protein measurement

Unfolded protein content was measured in hippocampal lysate and enriched mitochondrial fraction. 10 μg of protein was diluted to 100 μL of PBS and incubated with 40 μM of TPE-MI (catalog no. HY-143218, MedChemExpress, USA). Fluorescence was measured in BioTek Synergy HT (360 nm, 470 nm) [40].

### Immunoprecipitation

200 ug of proteins of the hippocampal total lysate were incubated with 10 μL of agarose beads for 30 min in agitation at 4 °C. The sample was centrifuged at 3000 rpm for 2 min, the supernatant was collected and incubated with 0,2 µg of the control IgG antibody, or the interest IgG antibody (Lonp1) at 4 °C in orbital agitation overnight. 20 µl of agarose beads were added and incubated for 1 h at 4 °C in orbital agitation. The sample was centrifuged at 3,000 rpm for 2 min at 4 °C, and the supernatant was discarded. The pellet was resuspended in PBS 1x, centrifuged at 3,000 rpm for 2 min, and then resuspended again in PBS 1x [41].

### FITC-Casein Lonp1 proteolytic activity assay

Immunoprecipitated Lonp1 samples were centrifuged at 3,000 rpm for 2 min at 4 °C, and the pellet was resuspended in activity buffer (150 mM NaCl, 50 mM HEPES-KOH pH 8.0, 10 mM MgCl₂, 0.1 mg/mL BSA) containing FITC-casein (0.1 mg/mL) and ATP (2 mM). The degradation kinetics of FITC-casein were measured using the Pierce™ Fluorescent Protease Assay Kit at 485–538 nm in a BioTek Synergy plate reader. Measurements were taken every 15 s for 1 h in C57BL/6 J samples, while in Senescence-Accelerated Mouse Prone 8 (SAMP8) samples the measurements were recorded up to 30 minutes.

### Image analysis

Densitometry analysis for immunoblot images was performed using ImageJ (NIH) software, and the data were normalized to a specific loading control, as indicated in each figure legend. Confocal images were acquired under the same magnification, laser intensity, brightness, and gain. Images were processed using the Fiji software (NIH Image), adjusting the fluorescence threshold intensity in every picture [28, 37].

### Statistical analysis

All data are expressed as mean ± standard error of the mean (SEM). We used two-way ANOVAs to determine the effects of sex, age, and sex-by-age interaction. Where appropriate, Tukey’s post-hoc comparisons were performed to examine the individual main effect of sex and age. The assumptions of normally distributed data and homogeneous variances were confirmed using Shapiro-Wilk and Levene’s tests, respectively. Statistical analyses were performed using GraphPad Prism 8 (San Diego, CA, USA) and Statistica (StatSoft Tulsa, OK) software packages. Differences were considered statistically significant at *p*-values ≤ 0.05. *p*-values between 0.01 and 0.05 are indicated with one asterisk, *p*-values between 0.001 and 0.01 with two asterisks, *p*-values between 0.001 and 0.0001 with three asterisks, and *p*-values less than 0.0001 with four asterisks. Investigators were not blinded to allocation during the experiments, use of animals or outcome assessment. For animal studies and experiments with animal derived tissues or samples no randomization was used.

## Results

### Hippocampal-dependent cognitive decline is differentially displayed in aged male and female mice

**Fig. 1:**
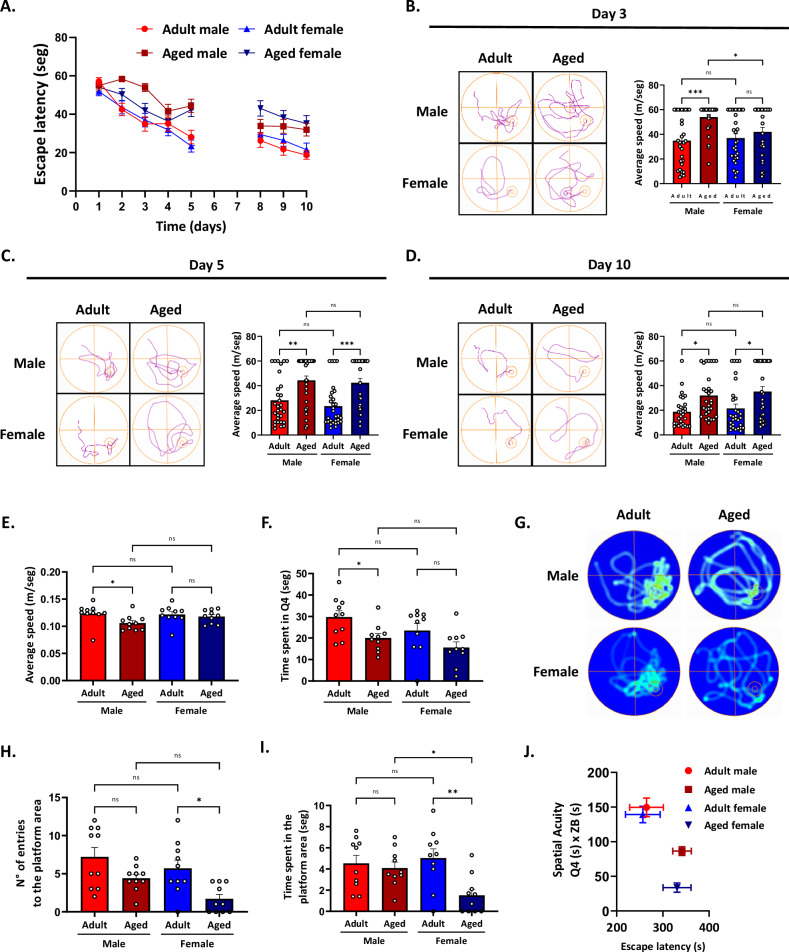
Spatial memory impairment is sexually dimorphic in aged mice. Behavioral performance was assessed using the Morris Water Maze (MWM) test. **A** Escape latency (time to reach the hidden platform) was recorded in 10 days of the test for male/female adult and aged mice. **B**-**D** Representative swimming trajectories and escape latency for all experimental groups on days 3, 5, and 10. **E** Average swimming speed. **F** Analysis of the time mice spent swimming in the quadrant where the platform was located (probe trial). **G** Representative heat maps of the probe trial on day 11. **H** Number of entries to the platform area in the probe trial on day 11. **I** Time mice spent in the platform area in the probe trial on day 11. **J** Spatial acuity for male/female adult and aged mice. Spatial acuity was calculated using data from the learning phase and the probe trial (see methods). Values represent means ± SEM. Statistical differences were calculated by two-way ANOVA. *p < 0.05, **p < 0.01, ***p < 0.001.

Aging is associated with cognitive decline among mammal species [42]. Specifically, the hippocampus, a brain structure known for its functions related to learning and spatial memory, recognition memory, and the consolidation of short and long-term memory, undergoes a progressive decline in function with age [43–45], but whether this decline depends on sex is not entirely understood. We initially tested spatial memory using the Morris Water Maze (MWM) task, which involves training mice to locate a hidden platform based on external cues [13]. During the training phase, both adult male and female mice exhibited a gradual and progressive learning curve, with their performance improving over time as they learned the location of the platform. In contrast, aged mice delayed learning the platform location in a sex-dependent manner. In the early days of the test, aged males learned the platform location more slowly than aged females. However, as the test progressed, aged males eventually achieved an escape latency comparable to that of aged females. This suggests that, although their learning process is initially slower, they can ultimately learn the task (Fig. 1A). We further analyzed the escape latency in several days. On day 3 of the test, the two-way ANOVA analysis of the time taken to reach the platform showed no significant effect of sex (*p* = 0.14) but a significant effect of age [F_(1,116)_ = 13.15; *p* < 0.01], and there was a significant interaction between both factors [F_(1,116)_ = 4.48; *p* = 0.03]. Specific analysis revealed that aged males took significantly longer to reach the platform than adult males. In addition, aged males showed longer escape latencies to reach the platform than aged females, suggesting that aged males have more difficulty learning the platform location (Fig. 1B). On days 5 and 10 of the test, the two-way ANOVA analyses showed no significant effect of sex (day 5, *p* = 0.32 and day 10, *p* = 0.37), a significant effect of age [day 5, F_(1,116)_ = 26.28; *p* < 0.001 and day 10, F_(1,116)_ = 16.29; *p* < 0.001], and there was no significant interaction between the two factors (day 5, *p* = 0.69 and day 10, *p* = 0.93). Specific analysis showed that the time taken to reach the platform was higher in aged animals compared to adult animals. Similarly, aged females took longer to reach the platform than adults (Fig. 1C, D), indicating a persistent learning impairment with aging. We also assessed the motor capacity of the mice over the 10-day test period. The statistical analysis revealed no significant effect of sex (*p* = 0.31), a significant effect of age [F_(1,36)_ = 4.75; *p* = 0.03), but was not affected by the interaction between the two factors (*p* = 0.18). Specific analysis showed that aged animals were slower than adult ones (Fig. 1E). These findings suggest that aging affects primarily the initial task acquisition, possibly due to short-term memory deficits in aged males, who exhibit significantly longer escape latency in the early days of testing compared to both adults and females. However, as the test progressed, aged males eventually reached a similar latency to that of aged females, indicating that, despite initial differences, long-term memory remains comparable between the two aged groups. Additionally, it is important to consider that aged males present motor deficiencies in swimming speed, which could influence their performance. However, the learning curve patterns suggest that cognitive factors play a central role in these differences.

Next, to evaluate spatial memory, on day 11 of the memory task, we conducted a probe test in which the platform was removed. This test assesses various parameters, including the time spent in the target (i.e., the area where the platform was located) [46]. Two-way ANOVA analyses on the Time in Q4 showed no significant effect of sex (*p* = 0.06), a significant effect of age [F_(1,36)_ = 10.34; *p* = 0.027], and there was no significant interaction between the two factors (*p* = 0.73). Specific analysis indicated that the Time in Q4 was lower in aged animals compared to adult ones (Fig. 1F), as illustrated in the representative heatmaps (Fig. 1G). Next, we analyzed the number of entries to the platform area (i.e., a circular area twice the platform’s radius). The statistical analysis revealed a significant effect of sex [F_(1,36)_ = 5.20; *p* = 0.03] and a significant effect of age [F_(1,36)_ = 13.86; *p* < 0.001], but was not affected by the interaction between the two factors (*p* = 0.51). Specific analyses revealed that females made fewer entries into the platform area than males. In terms of age, aged animals also showed fewer entries compared to adults (Fig. 1H). We then analyzed the time spent in the platform area and found no significant effect of sex (*p* = 0.15) but a significant effect of age [F_(1,36)_ = 7.99; *p* = 0.008], and a significant interaction between the sex and age [F_(1,36)_ = 4.83; *p* = 0.003]. Notably, we observed a sex-dependent effect, where aged females spent significantly less time in this area compared to adult females and aged males (Fig. 1I). These results suggest that, after learning the platform location, aged males outperform their female counterparts in the probe trial, which assesses reference memory. To further investigate spatial acuity, a more sensitive measure of spatial learning, we analyzed the probability of mice locating themselves in the specific region where the platform was previously placed. Figure 1J illustrates the relationship between spatial acuity and the escape latency in the MWM test. The statistical analysis of spatial acuity revealed no significant effect of sex (*p* = 0.21), but a significant effect of age [F_(1,36)_ = 11.60; *p* < 0.01], with no significant interaction between factors (*p* = 0.39). Similarly, the analysis of escape latency showed no effect of sex (*p* = 0.82), a significant effect of age [F_(1,36)_ = 12.52; *p* < 0.01], and no interaction between both factors (*p* = 0.80). Further analysis revealed that aged mice of both sexes exhibited lower spatial acuity and increased escape latency compared to adult mice (Fig. 1J). Our findings reveal sex-dependent differences in how aging affects spatial memory processes. Aged males showed greater short-term memory deficits, learning the task more slowly than aged females. However, long-term memory impairment is comparable in both aged groups. Notably, in the probe trial assessing baseline memory performance, aged males outperformed females, spending more time in the platform area and demonstrating better spatial acuity. Although the slower swim speed of aged males may contribute to their initial longer escape latency, cognitive factors appear to play a key role in these differences.

**Fig. 2:**
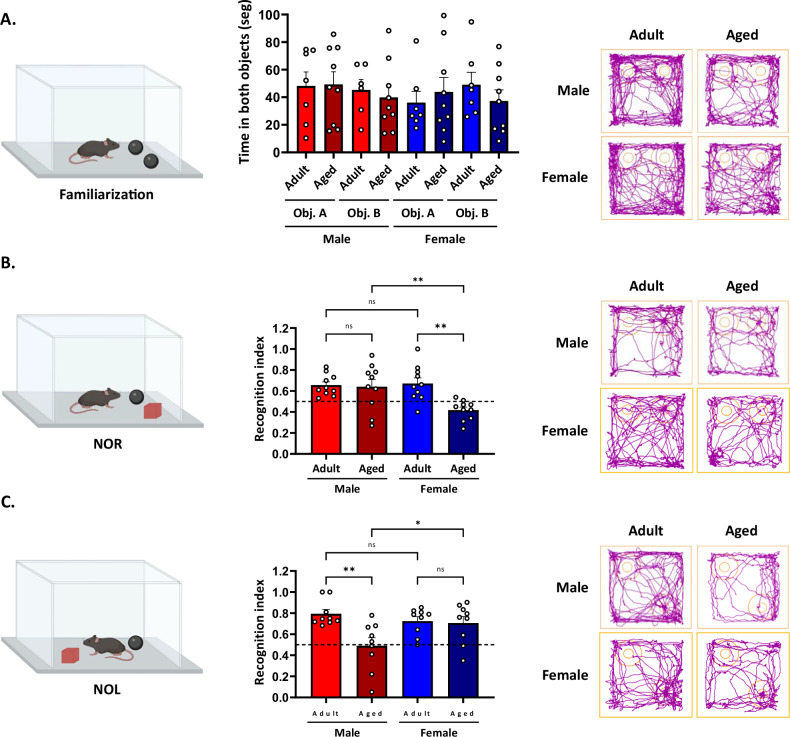
Recognition memory is differentially impaired between males and females during aging. Behavioral performance was evaluated using the NOR and NOL tests. The left panel visually represents the test phase; the middle panel shows the recognition index for NOR and NOL tests, and the right panel shows representative track plots. **A** The familiarization phase showed no discrepancies in object exploration. **B** NOR phase shows that aged females had no preference for the novel object, while (**C**) NOL phase shows that aged males had no preference for the novel localization of the object. Values represent means ± SEM. Statistical differences were calculated by two-way ANOVA. *p < 0.05, **p < 0.01.

Recognition memory mainly depends on the hippocampus and undergoes age-related alterations [47–49]. In this study, we examine how aging affects recognition memory in a sex-dependent manner. We tested recognition and localization memory using the Novel Object Recognition (NOR) and Novel Object Location (NOL) tests. Before both tests, mice underwent a familiarization phase, during which they explored two identical objects (object A and object B; Fig. 2A, left panel). Statistical analysis showed no significant effect of the object (males: *p* = 0.43 and females: *p* = 0.69), age (males: *p* = 0.78 and females: *p* = 0.80), or their interaction (males: *p* = 0.67 and females: *p* = 0.24). All experimental groups spent similar amounts of time exploring both objects (Fig. 2A, middle panel). Two hours later, the NOR test was performed, during which mice were presented with both a familiar and a novel object. The two-way ANOVA analyses on the Recognition Index showed a significant effect of sex [F_(1,36)_ = 4.39; *p* = 0.04], a significant effect of age F_(1,36)_ = 7.540; *p* = 0.009], and there was a significant interaction between both factors [F_(1,36)_ = 5.96; *p* = 0.02]. Both adult groups preferred the novel object; however, only aged female mice exhibited an impaired object recognition index, compared to adult females (Fig. 2B), indicating that aged females, but not aged males, exhibit impaired object recognition abilities. Two hours after the NOR test, we relocated the novel object inside the chamber to test object localization (NOL). In contrast to the NOR results, the two-way ANOVA analyses on the recognition index showed no significant effect of sex (*p* = 0.22), a significant effect of age [F_(1,32)_ = 7.76; *p* < 0.01], and a significant interaction between both factors [F_(1,32)_ = 6.12; *p* = 0.02]. Specific analysis revealed that only aged male mice exhibited a reduced localization recognition index, whereas aged females preferred the new localization of the object (Fig. 2C). These results suggest that aging affects recognition memory in a sex-dependent manner. Aged females exhibit impairments in object recognition, whereas aged males display deficits in object location memory.

We further investigated whether aging affected locomotion and anxiety-like behavior. Using the Open Field (OF) test, which evaluates both parameters, we found no significant differences between adult males and female mice across all measured variables (Fig. S1). The statistical analysis of the distance traveled showed no significant effect of sex (*p* = 0.85), age (*p* = 0.53), or their interaction (*p* = 0.44). Likewise, walking speed did not differ significantly concerning sex (*p* = 0.83), age (*p* = 0.50), or their interaction (*p* = 0.45). Then, we analyzed the number of entries into the center of the arena, a proxy for anxiety-like behavior. Again, no significant effects of sex (*p* = 0.45), age (*p* = 0.81), or interaction between the two factors (*p* = 0.66) were detected.

Our findings demonstrate that aging differentially affects hippocampal-dependent memory in male and female mice, affecting short- and long-term spatial memory, recognition memory, and localization memory. Regarding spatial learning, aged males exhibited more significant deficits in short-term memory acquisition than aged females; however, this difference was not observed in long-term memory. Despite initial learning impairment, aged males outperformed aged females in spatial memory, spending more time in the target area and showing better spatial acuity. Similarly, aged males showed deficits in object localization memory, whereas aged females showed impairments in object recognition memory. These findings underscore the complexity of cognitive aging and highlight the importance of investigating the molecular and cellular mechanisms underlying these sex-dependent differences.

### Mitochondrial oxidative stress is more severe in aged male mice than in aged female mice

**Fig. 3:**
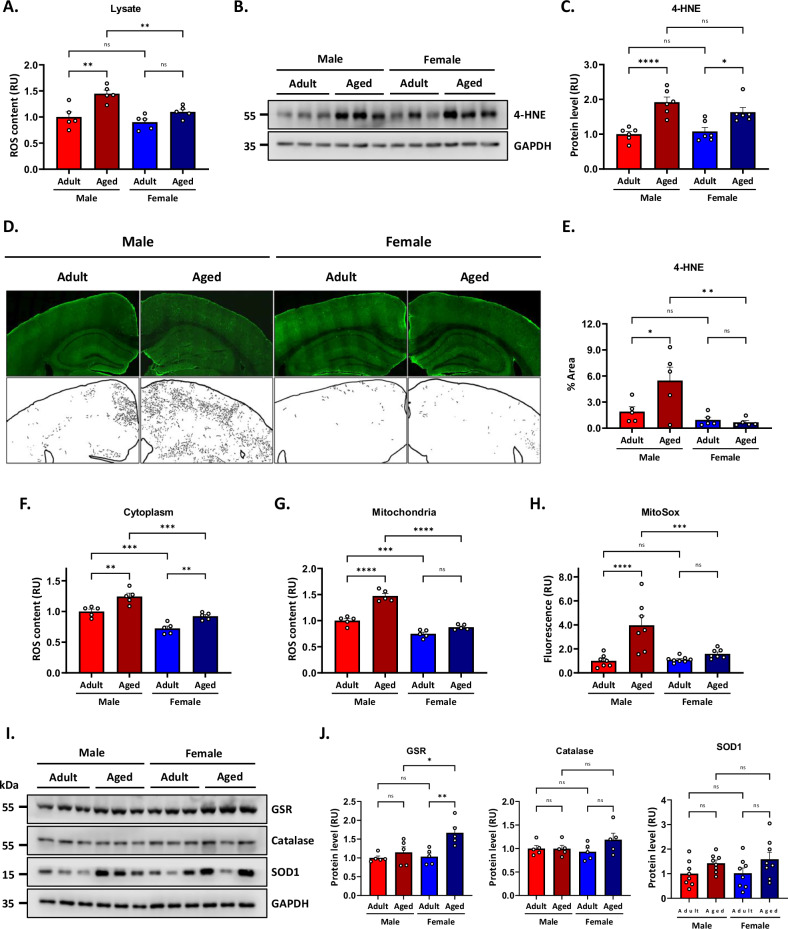
Oxidative stress is exacerbated in aged male mice. **A** ROS content in the hippocampal protein samples, measured by the fluorescent dye CM-H2DCFDA. **B** Western blot analysis for 4-hydroxynonenal (4-HNE) in hippocampal lysate with their (**C**) densitometric analysis expressed relative to the adult male group. **D** Immunofluorescence of the oxidative damage marker 4-hydroxynonenal (4-HNE) in brain slices with their respective segmentation and (**E**) quantification. ROS content in the hippocampal (**F**) cytoplasmic and (**G**) mitochondria-enriched fractions was measured by the fluorescent dye CM-H2DCFDA. **H** Superoxide production of an isolated mitochondrial-enriched fraction measured by the fluorescent dye MitoSox. **I** Western blot assay for antioxidant enzymes in hippocampal protein samples. Glutathione Reductase (GSR), Catalase, and Superoxide dismutase 1 (SOD1) were analyzed through densitometric analysis (**J**). Data is expressed relative to the adult male group. Values represent means ± SEM. Statistical differences were calculated by two-way ANOVA. *p < 0.05, **p < 0.01, ***p < 0.001, ****p < 0.0001.

Oxidative stress is a major feature of aging due to the increased production of reactive oxygen species (ROS) that damage DNA, lipids, and proteins [29]. In aging, specifically in the hippocampus, increased mitochondrial ROS production has been correlated with memory impairment [13]. Given that aged males showed greater memory decline than aged females, and we previously described a redox imbalance in the female hippocampus [28], we aimed to investigate sex-dependent differences in hippocampal ROS levels. For this, we first prepared protein samples from the hippocampus of both adult and aged mice of both sexes. We then analyzed ROS content in the four experimental groups using the fluorescent dye DCF CM-H2DCFDA, which oxidizes in the presence of different reactive oxygen species, emitting fluorescence proportional to the ROS levels in the sample [28]. The two-way ANOVA analyses of ROS content in total lysates revealed a significant effect of sex [F_(1,16)_ = 9.59; *p* < 0.01], and age [F_(1,16)_ = 20.04; *p* < 0.01], with no significant interaction between the two factors (*p* = 0.10). Aged male mice exhibited elevated ROS levels, as indicated by a higher fluorescent signal than adult males and aged females (Fig. 3A). Next, we evaluated oxidative damage using 4-HNE, an oxidative marker resulting from lipid peroxidation (Fig. 3B, C). Consistently, the statistical analyses of 4-HNE protein levels revealed a non-significant effect of sex (*p* = 0.41) but a significant effect of age [F_(1,20)_ = 35.46; *p* < 0.01], and no significant interaction between the two factors (*p* = 0.16). Specific analysis showed that aged animals exhibited higher levels of 4-HNE protein adducts than adults. This was determined by immunoblot analysis of total hippocampal lysates (Fig. 3B, C). This was further analyzed by immunofluorescence, followed by segmentation analysis in brain slices (Fig. 3D, E).

The analysis revealed a significant effect of sex [F_(1,16)_ = 11.49; *p* < 0.003], no significant effect of age (*p* = 0.07), and a significant interaction between both factors [F_(1,16)_ = 5.15, *p* = 0.04].

Post hoc analyses showed that aged males exhibited higher 4-HNE protein levels compared to both adult males and aged females. Mitochondria are the primary producers of ROS during aging [50]. Therefore, as a first approach, we measured ROS content in the mitochondria-free cytoplasm and in a mitochondrial fraction from the hippocampus to assess whether the observed increase in ROS content could be associated with mitochondrial activity or derived from other cellular sources. The two-way ANOVA analyses on the cytoplasmic fraction showed a significant effect of sex [F_(1,16)_ = 53.93; *p* < 0.01], a significant effect of age [F_(1,16)_ = 29.77; *p* < 0.01], but was not affected by the interaction between the two factors (*p* = 0.61). Further analysis indicated that the cytoplasmic fraction of aged male mice presented higher ROS levels compared to female mice (Fig. 3F), in agreement with the results observed in the total hippocampal lysate (Fig. 3A). Statistical analysis showed a significant effect of sex [F_(1,16)_ = 9.57; *p* < 0.01], and significant effect of aged [F_(1,16)_ = 20.04; *p* < 0.01], with no significant interaction between the two factors (*p* = 0.10). Similarly, adult and aged females displayed lower ROS content than adult and aged males (Fig. 3F). Subsequently, mitochondrial ROS levels were analyzed, showing a comparable pattern. There was a significant effect of sex [F_(1,16)_ = 133.0; *p* < 0.01], a significant effect of age [F_(1,16)_ = 66.31; *p* < 0.01], and a significant interaction between both factors [F_(1,16)_ = 21.88; *p* < 0.01]. Interestingly, the female group does not exhibit changes in ROS content with aging (Fig. 3G), supporting the notion that aged males exhibit more severe oxidative stress at cellular and mitochondrial levels, differences that are evident from adulthood and increase with age.

Mitochondria generate ROS as sub-products of the electron transport chain (ETC). The main ROS are superoxide anion (O₂-), generated by the leakage of electrons in complexes I and III [51]. Since we observed that mitochondrial ROS content increased in aged males but not females, we assessed mitochondrial superoxide production to determine whether this difference could be driving the observed sex-specific ROS levels during aging. To test this, we obtained a hippocampal mitochondria-enriched fraction from adult and aged animals. We incubated them with oxidative substrates and the fluorescent dye MitoSox, where their fluorescence is proportional to mitochondrial superoxide production by OXPHOS function. The statistical analyses revealed a significant effect of sex [F_(1,28)_ = 10.07; *p* < 0.01], a significant effect of age [F_(1,28)_ = 23.14; *p* < 0.01], and a significant interaction between both factors [F_(1,28)_ = 11.95; *p* < 0.01]. Surprisingly, superoxide production was four times higher in aged males and exceeded that of aged females, who did not exhibit a significant increase in superoxide levels compared with adult females (Fig. 3H). These results strongly suggested that increased oxidative damage in aged males is caused, almost partly, by an exacerbated mitochondrial ROS production.

When the superoxide ion is produced, it is rapidly converted to hydrogen peroxide (H₂O₂) by the enzyme superoxide dismutase (SOD) as part of the antioxidant response [52]. We previously showed that aged female mice had increased levels of the antioxidant proteins glutathione reductase (GSR) and superoxide dismutase 1 (SOD1) [28]. Here, we evaluated GSR, catalase, and SOD1 enzymes in the aging process of male and female mice (Fig. 3I). We found that the two-way ANOVA analyses on the GSR protein levels showed a significant effect of sex [F_(1,16)_ = 5.55; *p* = 0.03], a significant effect of age [F_(1,16)_ = 11.0; *p* = 0.004], but was not affected by the interaction between the two factors (*p* = 0.06). The levels of GSR protein were elevated in the hippocampus of aged female mice, while no changes were observed in aged males (Fig. 3J). In contrast, catalase levels showed no significant effects of sex (*p* = 0.51), age (*p* = 0.18), or their interaction (*p* = 0.17), suggesting that catalase levels remain unchanged across both aged groups (Fig. 3J). For SOD1, the two-way ANOVA revealed no significant effect of sex (*p* = 0.67) but a significant effect of age [F_(1,28)_ = 5.65; *p* = 0.02], with no interaction between the two factors (*p* = 0.73). However, post-hoc analysis indicated that SOD1 remained unchanged across sex and age groups.

These results showed that aged males have more severe oxidative stress than the adult groups and aged females. The antioxidant protein levels results suggest that the antioxidant response might be more active in females at an advanced age, highlighting the sex-related differences in oxidative stress in the aged hippocampus and implying that the difference in redox balance could contribute to differential memory impairment in aging.

### ATP deficiency is more severe in aged male mice

**Fig. 4:**
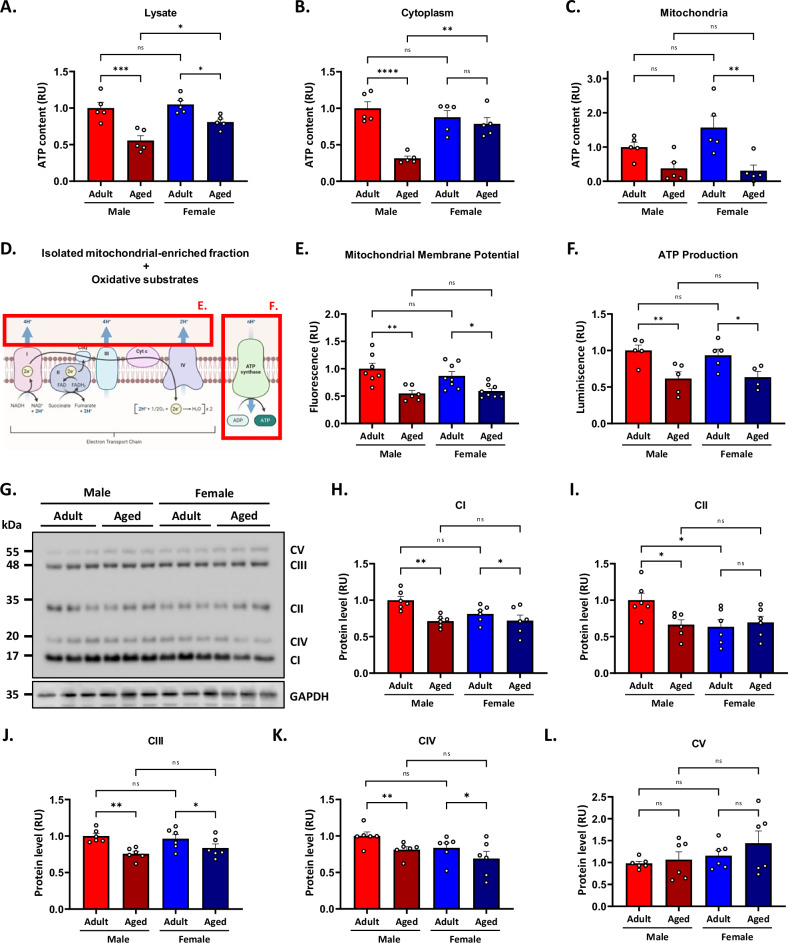
Mitochondrial bioenergetic impairment is more severe in aged male mice. ATP content was measured in hippocampal lysate (**A**), (**B**) cytoplasmic, and (**C**) mitochondrial fractions using an ATP bioluminescence detection kit. **D** Representative scheme of the electron transport chain and protocol to measure mitochondrial function. Mitochondrial membrane potential (**E**) and ATP production (**F**) were measured in an enriched mitochondrial fraction exposed to oxidative substrates. **G** Western blot analysis of oxidative phosphorylation protein complex (OXPHOS) levels. **H–L** Densitometric analysis of OXPHOS complexes is expressed as levels relative to the aged male groups. Values represent means ± SEM. Statistical differences were calculated by two-way ANOVA, *p < 0.05, **p < 0.01, ***p < 0.005, ***p < 0.001, ****p < 0.0001.

The primary mitochondrial function involves the production of ATP, and it is known that during aging, mitochondrial function and ATP formation are reduced in the hippocampus [13, 28]. However, whether sex-dependent differences emerge during aging remains unclear. First, we evaluated the bioenergetic state of the hippocampus. To this end, whole hippocampal lysate, mitochondria-free cytoplasmic, and mitochondrial-enriched fractions were tested using a bioluminescence detection kit to measure the ATP content. The two-way ANOVA analyses revealed a significant effect of sex [F_(1,16)_ = 6.00; *p* = 0.03] and a significant effect of age [F_(1,16)_ = 30.542; *p* < 0.01], with no significant interaction between the two factors (*p* = 0.12). Both aged groups exhibited a reduction in ATP content compared to adult mice, with a more pronounced decrease in aged male mice than in the female ones (Fig. 4A). More importantly, in the cytoplasmic fraction, the analysis showed a significant effect of sex [F_(1,16)_ = 4.96; *p* = 0.04], a significant effect of age [F_(1,16)_ = 24.42; *p* < 0.01], and a significant interaction between both factors [F_(1,16)_ = 14.47; *p* = 0.001]. Further analysis revealed that aged males exhibited approximately a 70% reduction in cytoplasmic ATP content, whereas females showed no changes during aging (Fig. 4B). On the other hand, the mitochondrial fraction showed no significant effect of sex (*p* = 0.27), but a significant effect of age [F_(1,16)_ = 18.25; *p* < 0.01], with no significant interaction between the two factors (*p* = 0.16). Specific analysis showed that adult animals had higher ATP content mitochondria than the aged ones (Fig. 4C). These results suggest that the bioenergetic state of the hippocampus differs between aged males and females; aged males present reduced energy in the cytoplasm whereas aged females have reduced levels of ATP in the mitochondria. Then, we tested the functionality of the electron transport chain of isolated hippocampal mitochondria in the different experimental groups by measuring the mitochondrial membrane potential and the ATP production (Fig. 4D). For this, mitochondrial enriched fractions were incubated with oxidative substrates and loaded with the fluorescent dye MitoTracker Red CM-H2Xro, which accumulates in the mitochondrial matrix driven by the electrochemical gradient generated by the electron transport chain; this makes it sensitive to the mitochondrial membrane potential, allowing it to detect active mitochondria, since its fluorescence depends directly on this potential. Interestingly, the statistical analyses for mitochondrial membrane potential and ATP production revealed no significant effect of sex (mitochondrial membrane potential: *p* = 0.51; ATP production: *p* = 0.27) but a significant effect of age [mitochondrial membrane potential: F_(1,28)_ = 30.40; *p* < 0.01 and ATP production: F_(1,16)_ = 18.25; *p* < 0.01]. No significant interaction between sex and age was observed (mitochondrial membrane potential: *p* = 0.20; ATP production: *p* = 0.16).

Specific analysis showed that both mitochondrial membrane potential and ATP production of aged animals had lower mitochondrial membrane potential values than adult individuals (Fig. 4E, F). In addition, and searching for a possible explanation for the observed mitochondrial dysfunction, we evaluated the protein levels of mitochondrial respiratory chain complexes involved in oxidative phosphorylation (OXPHOS) using the antibody cocktail OXPHOS complexes [36] (Fig. 4G). For complex I, we found no significant effect of sex (*p* = 0.11), and a significant effect of age [F_(1,20)_ = 12.04; *p* < 0.01], with no significant interaction between the two factors (*p* = 0.09). Aged animals showed lower values of complex I. For complex II, no significant main effects of sex (*p* = 0.07) or age (p = 0.13) were observed. However, a significant interaction between sex and age was detected [F_(1,20)_ = 5.23; *p* = 0.03]. Post hoc analysis revealed that adult males exhibited higher complex II protein levels. For complex III, we found no significant effect of sex (*p* = 0.27), a significant effect of age [F_(1,20)_ = 9.79; *p* = 0.005], and no significant interaction between the two factors (*p* = 0.09). Specific analysis indicated that Complex III levels was lower in aged animals compared to adult ones. For Complex IV, there was no significant effect of sex (*p* = 0.05), but a significant main effect of age was observed [F_(1,20)_ = 17.02; *p* = 0.005]. No significant interaction between sex and age was detected (*p* = 0.12). Post hoc analysis revealed that aged animals exhibited lower Complex IV than adults. For Complex V, there were no significant effects of sex (*p* = 0.14), age (*p* = 0.31), or their interaction (*p* = 0.59) (Fig. 4H–L). Thus, our findings reveal sex-dependent differences in hippocampal bioenergetics during aging, highlighting distinct vulnerabilities in ATP production and mitochondrial function. Aged males present decreased cytoplasmic ATP content, whereas aged females show reduced mitochondrial ATP content, suggesting differential energy deficits between sexes.

Also, although both aged groups show deficient mitochondrial membrane potential and ATP production, the decline is more pronounced in males, and this could be due, almost in part, to a significant reduction in the protein levels of mitochondrial respiratory complexes I-IV. These results also reveal a sex difference since adulthood, where adult males exhibit increased protein levels of complex I, II, and IV compared to adult females. These results suggest sex differences in mitochondrial function, even in adults, and that aging affects mitochondrial function and energy metabolism differently in males and females, which may contribute to the observed sex-dependent cognitive impairments.

### mPTP activity is increased in aged female mice

**Fig. 5:**
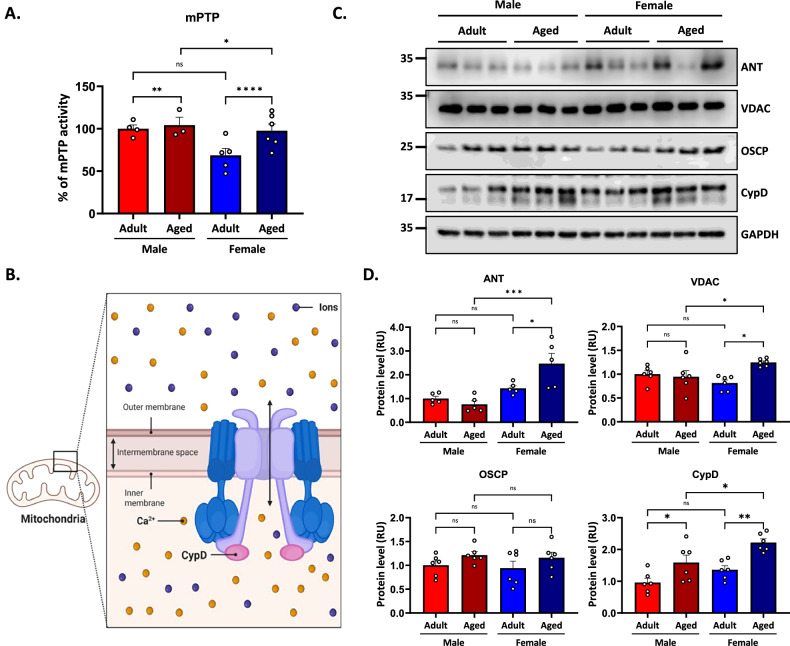
Mitochondrial permeability transition pore (mPTP) activity increases in aging and is more pronounced in aged females. **A** Measurement of mPTP activity in isolated mitochondria through a fluorescent mPTP Assay. **B** Representative scheme of the mitochondrial permeability transition pore (mPTP). **C** Western blot analysis of hippocampal lysates for the protein components of the mPTP: ANT, VDAC, OSCP, and CypD. **D** Densitometric analysis is expressed as levels relative to the control. Values represent means ± SEM. Statistical differences were calculated by two-way ANOVA, *p < 0.05, **p < 0.01, ***p < 0.001, ****p < 0.0001.

Calcium homeostasis is essential for cell survival. The calcium balance must be strictly regulated to maintain functional mitochondria [53] because mitochondria serve by buffering high calcium levels [36]. However, when mitochondria are unable to regulate calcium overload, they undergo swelling and promote the opening of the mitochondrial permeability transition pore (mPTP), resulting in cell death [36]. We recently described increased mPTP activity in aged female mice [28]. Considering that mitochondria from aged males exhibit higher oxidative stress than those from females, and that elevated ROS can trigger mPTP opening, we wondered whether males display differential mPTP activity during aging. mPTP openings were measured in a mitochondrial fraction enriched using the mPTP assays; this assay employs Calcein-AM to accumulate in live cell mitochondria, and Cobalt (Co^2+^) acts as a quencher, diminishing calcein’s fluorescence when the mPTP remains open over time. We evaluated the opening of the mPTP in all our experimental groups (Fig. 5). We found that mPTP activity on hippocampal mitochondria revealed no significant effect of sex (p = 0.112) but a significant effect of age [F_(1,24)_ = 47.89; *p* < 0.01], and there was a significant interaction between both factors [F_(1,24)_ = 5.19; *p* = 0.03]. Further analysis indicated higher values in the aged groups; however, surprisingly, and contrary to bioenergetic results, aged females had a more active mPTP than aged males (Fig. 5A). Several factors can contribute to the prolonged opening of mPTP, such as excessive calcium in the mitochondria matrix, the imbalance between free radicals and antioxidants leading to oxidative damage, and loss of mitochondrial membrane potential [54, 55]. Another critical point is the molecular identity of the mPTP. The levels of protein components of this mitochondrial pore in adults and aged mice, as well as evaluating sex differences, remain challenging to date. For this motive, we assessed the proteins more recognized as members of mPTP [55, 56], such as ANT (Adenine nucleotide translocase) protein situated in the inner mitochondrial membrane, VDAC (Voltage-dependent anion channel), a protein found in the outer mitochondrial membrane, OSCP (Oligomycin sensitivity conferral protein), a subunit of mitochondrial ATP synthase, and CypD (Cyclophilin D) protein found in the mitochondrial matrix (Fig. 5B) [57]. We aimed to determine whether sex-related differences in aging alter mPTP component levels. Western blot analysis was performed on hippocampal lysates from adult and aged mice of both sexes (Fig. 5C). The two-way ANOVA for ANT levels revealed a significant effect of sex [F_(1,16)_ = 19.67; *p* < 0.01], no significant effect of age (*p* = 0.12], and a significant interaction between both factors [F_(1,16)_ = 7.12; *p* = 0.02]. Further analysis indicated that aged females had higher levels than adult females, with aged females showing an increase in ANT levels compared to aged males (Fig. 5D). For VDAC levels, no significant effect of sex was detected (*p* = 0.49), while a significant effect of age [F_(1,20)_ = 5.26; *p* = 0.03] and a significant sex × age interaction [F_(1,20)_ = 8.69; *p* = 0.007] were observed. Post hoc analysis revealed a significant increase in VDAC levels in aged females compared to both adult females and aged males (Fig. 5D). For OSCP levels no significant effect of sex (*p* = 0.60), age (*p* = 0.06), or interaction between the two factors (*p* = 0.99) was found. Finally, CypD levels showed a significant effect of sex [F_(1,20)_ = 10.45; *p* < 0.01], a significant effect of age [F_(1,20)_ = 21.55; *p* < 0.01], but were not affected by the interaction between the two factors (*p* = 0.48). Our findings indicate that mitochondrial calcium homeostasis is differentially affected by aging, depending on sex. Although aging males and females exhibit higher mPTP activity, females display a more pronounced opening of mPTP, suggesting a greater susceptibility to mitochondrial calcium fluctuations and/or overload. This could be partly explained by the increase in the expression of mPTP-associated proteins ANT, VDAC, and OSCP, whereas the rise in mPTP activity in aged males could also be related to the increase in CypD. Altogether, these results suggest that distinct molecular mechanisms underlie mPTP regulation in aging, with females potentially being more dependent on mPTP components that facilitate pore opening.

### Mitochondrial protein quality control failure in the aged hippocampus of mice

**Fig. 6:**
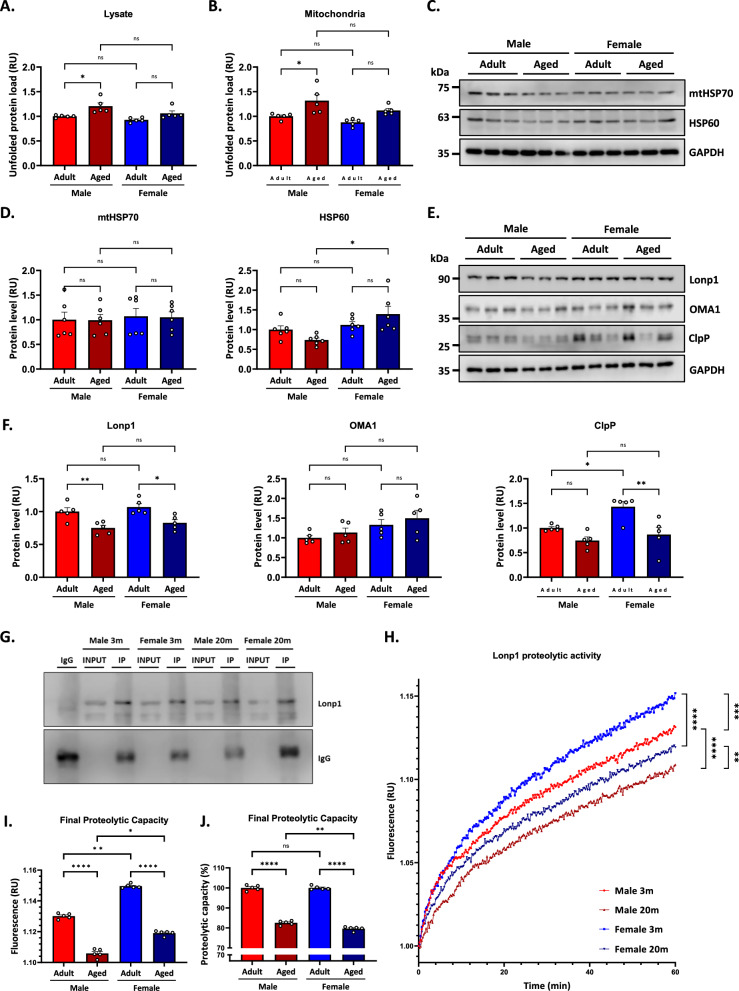
Higher mitochondrial protein quality control failure in aged male mice. **A** The unfolded protein load was measured in a hippocampal protein sample using TPE-MI. **B** The unfolded protein load was measured in a hippocampal enriched mitochondrial fraction using TPE-MI. **C** Western blot analysis of mitochondrial chaperones mtHSP70 and HSP60 levels in a hippocampal lysate. **D** Densitometric analysis of chaperones is expressed as levels relative to the aged male groups. **E** Western blot analysis of mitochondrial proteases Lonp1, OMA1, and ClpP levels in a hippocampal lysate. **F** Densitometric analysis of proteases is expressed as levels relative to the aged male group. **G** Representative immunoblots show the immunoprecipitated (IP) and input fractions probed for Lonp1 and IgG. **H** Lonp1 proteolytic activity was evaluated using a FITC-casein degradation assay, with fluorescence measurements taken every 15 s up to 60 min. Quantification of final proteolytic capacity expressed as (**I**) relative fluorescence units (RFU) and as (**J**) percentage of proteolytic capacity. Values represent means ± SEM. Statistical differences were calculated by two-way ANOVA, *p < 0.05, **p < 0.01, ***p < 0.001, ****p < 0.0001.

The maintenance of proteostasis is crucial for maintaining proper cellular function. Notably, an overall proteostasis imbalance occurs during aging [29, 58], but there is a lack of knowledge on what happens in the hippocampus, let alone possible differences between sexes. To study this, we evaluated the load of unfolded proteins using TPE-MI. This fluorescent dye interacts with free cysteines that do not form disulfide bridges, thus detecting unfolded or misfolded proteins [40]. We first measured the unfolded protein load using hippocampal lysates from all four experimental groups (Fig. 6A). Statistical analysis showed a significant effect of sex [F_(1,16)_ = 6.31; *p* = 0.03] and a significant effect of age [F_(1,16)_ = 14.82; *p* < 0.01], with no significant interaction between the two factors (*p* = 0.41). Specific analysis indicated that TPE total lysate was higher in males compared to females. In terms of age, aged animals also showed higher levels than adults (Fig. 6A). These results suggest that aging impairs cellular proteostasis in a sex-dependent manner.

Maintaining protein homeostasis within mitochondria is crucial for cellular function and overall health. Key players in this intricate system include chaperones and proteases that collaborate to ensure proper protein folding, the degradation of damaged proteins, and overall mitochondrial quality control [59]. Since we observed an increase in unfolded proteins exclusively in aged males when analyzing a total lysate, we assessed whether this increase was also present in mitochondria. To test this, all four experimental groups measured the unfolded protein load in enriched mitochondrial fractions. Statistical analyses revealed a significant effect of sex [F_(1,16)_ = 5.57; *p* = 0.03] and a significant effect of age [F_(1,16)_ = 16.83; *p* < 0.01], with no interaction between the two factors (*p* = 0.59). Specific analysis indicated that TPE mitochondria was higher in males compared to females. In terms of age, aged animals showed higher values compared to adults (Fig. 6B). Therefore, we measured the protein levels of essential components involved in the mitochondrial proteostasis machinery. We evaluated by immunoblotting the chaperones HSP60 and mtHSP70 (Fig. 6C, D) and the proteases Lonp1, ClpP, and Oma1 (Fig. 6E, F) to determine whether aging and sex influence their levels.

Two-way ANOVA analysis of mtHSP70 levels revealed no significant effect of sex (*p* = 0.63), age (*p* = 0.91), or their interaction (*p* = 0.96), indicating that mitochondrial mtHSP70 expression remains stable across both sex and age groups (Fig. 6C). In contrast,

HSP60 protein levels showed a significant effect of sex [F_(1,20)_ = 10.54; p = 0.004], with a non-significant effect of age (*p* = 0.96), but with a significant interaction between both factors [F_(1,20)_ = 5.07; *p* = 0.03]. We observed an increased HSP60 in aged females compared to aged males (Fig. 6D). For Lonp1, there was no significant effect of sex (*p* = 0.17), but a significant effect of age [F_(1,16)_ = 23.36; *p* < 0.01], and no significant interaction between the two factors (*p* = 0.92). Specific analysis showed that Lonp1 levels were lower in aged animals compared to adult ones (Fig. 6F). For OMA1, protein expression showed a significant effect of sex [F_(1,16)_ = 6.27; *p* = 0.02], but no significant effect of age (*p* = 0.29) or interaction (*p* = 0.91). Notably, OMA1 levels remained unchanged with aging in both sexes (Fig. 6G). Finally, ClpP levels showed a significant effect of sex [F_(1,16)_ = 7.57; *p* = 0.01] and age [F_(1,16)_ = 16.63; *p* < 0.01] but were not affected by the interaction (*p* = 0.14). Specific analysis showed that ClpP levels were lower in males than females. In terms of age, aged animals also showed higher levels than adults. This higher basal level of ClpP in adult females may attenuate the negative impact of proteostasis loss observed with aging (Fig. 6H). The results indicate that mitochondrial chaperones and proteases are differentially affected by aging in a sex-specific manner. Specifically, Lonp1 exhibits a more pronounced decrease in aged males, suggesting that specific mechanisms of mitochondrial proteostasis are selectively altered in aging in a sex-specific manner.

Lonp1 is a mitochondrial ATP-dependent protease responsible for degrading more than 50% of the mitochondrial proteome, including damaged, oxidized, or misfolded proteins, thereby maintaining mitochondrial proteostasis and ensuring proper organelle function [26]. Also, it has even been reported to degrade cytosolic proteins imported into the mitochondria [60]. After observing a more significant decrease in Lonp1 levels in aged males, we decided to assess whether its proteolytic activity also undergoes changes in aging and whether this is in a sex-dependent manner. First, immunoprecipitation of all experimental groups was performed to guarantee that the amount of immunoprecipitated Lonp1 was similar among the four groups (Fig. 6G), ensuring that the proteolytic activity assay was initiated with the same amount of protein and that the observed changes should be attributable exclusively to differences in proteolytic activity. Next, the proteolytic activity of Lonp1 was measured using a fluorescence-based assay with the fluorogenic substrate casein (FITC-Casein kit) under conditions of substrate and ATP saturation. This ensures that the observed differences in activity are not due to limitations in substrate availability but rather reflect inherent changes in the proteolytic capacity of Lonp1. We observed a reduction in proteolytic activity in both aged groups, with a more pronounced decline in aged males compared to aged females (Fig. 6H). The final proteolytic capacity of Lonp1, which is crucial for understanding its activity under low substrate concentrations and non-saturating conditions, was also assessed. The analysis indicated significant effect of sex [F_(1,16)_ = 343.3; *p* < 0.01], age [F_(1,16)_ = 964.5; *p* < 0.01], and a significant interaction between factors [F_(1,16)_ = 12.51; p < 0.01]. A decrease in final proteolytic capacity was observed across aging, with males mice exhibiting lower levels compared to females (Fig. 6I). When performing the same comparison between the adult groups and their aged counterparts, taking as reference 100% in adults, the statistical analysis indicated a significant effect of sex [F_(1,16)_ = 8.62; *p* < 0.01], age [F_(1,16)_ = 1475.00; *p* < 0.01], and a significant interaction [F_(1,16)_ = 8.62; *p* < 0.01]. Further analysis revealed a decrease in the final proteolytic capacity of Lonp1 in aging, with significant differences between aged males and females (Fig. 6J). This suggests that, since adulthood, females exhibit a higher proteolytic activity of Lonp1, which could explain why, in aging, they retain a higher proteolytic capacity compared to males, since they already possess a higher proteolytic activity in the adult stage.

Our findings reveal that aging alters proteostasis in the hippocampus in a sex-dependent manner, with elderly males showing a more significant accumulation of unfolded proteins than females. This age-related imbalance also extends to mitochondrial proteostasis, where aged males exhibit a more pronounced decrease in key proteases and chaperones compared to aged females, accompanied by a significant reduction in Lonp1 levels. Importantly, although Lonp1 expression decreases in both sexes with age, its proteolytic activity is more impaired in elderly males. This suggests that females may possess mechanisms that better preserve mitochondrial quality control during aging, such as higher basal levels of ClpP in aging and increased Lonp1 proteolytic activity from adulthood onwards. Thus, mitochondrial proteostasis appears to be more prone to failure in males than in females during aging.

Thus, our findings highlight significant sex-dependent differences in cognitive aging, with males exhibiting greater deficits in spatial and localization memory, while females show impairments in recognition memory. These cognitive differences are linked to distinct mitochondrial dysfunction mechanisms: aged males experience higher oxidative stress and ATP deficits, whereas aged females show increased mitochondrial permeability transition pore (mPTP) activity. Additionally, mitochondrial proteostasis is more prone to failure in aged males due to reduced Lonp1 protease activity. These results underscore the crucial role of sex in hippocampal aging and emphasize the need to consider sexual differences in aging research for a more comprehensive understanding of cognitive decline. Now, because sex hormones may influence both mitochondrial function and cognition, we ultimately assessed whether age-related hormonal changes might underlie the observed sex-specific differences in hippocampal aging. For this, we analyzed circulating levels of testosterone in males and estradiol in females. In males, testosterone levels were slightly lower in aged compared to adult individuals (GM in adult males: 0.33 ng/ml, 95% CI: 0.06–1.90; GM in old males: 0.29 ng/ml, 95% CI: 0.07–1.24), corresponding to a fold-change (Old/Adult) = 0.90, indicating an approximate 10% decline with age. In females, estradiol levels remained largely stable across age groups (GM in adult females: 5.63 pg/ml, 95% CI: 3.20–9.88; GM in old females: 5.48 pg/ml, 95% CI: 4.03–7.46), with a fold-change = 0.97, suggesting only a ~ 3% decrease. Statistical comparisons using Welch’s t-tests revealed no significant differences between age groups for either testosterone (t = 0.13, *p* = 0.90) or estradiol (t = 0.11, *p* = 0.91). Collectively, our results indicate that, within this sample analyzed, aging did not significantly alter basal circulating levels of testosterone in males or estradiol in females. However, a modest downward trend in testosterone with age may be present. Collectively, our data suggest that sex-specific cognitive and mitochondrial alterations during aging cannot be attributed to significant changes in basal gonadal hormones. Instead, they likely arise from intrinsic cellular and molecular mechanisms that differentially shape the aging of male and female brains. Future research should delineate the specific signaling pathways responsible for these sex-related differences and their relevance to neurodegenerative disorders associated with aging.

### SAMP8 mice recapitulate sex-specific mitochondrial dysfunction observed in C57BL/6 J mice

**Fig. 7:**
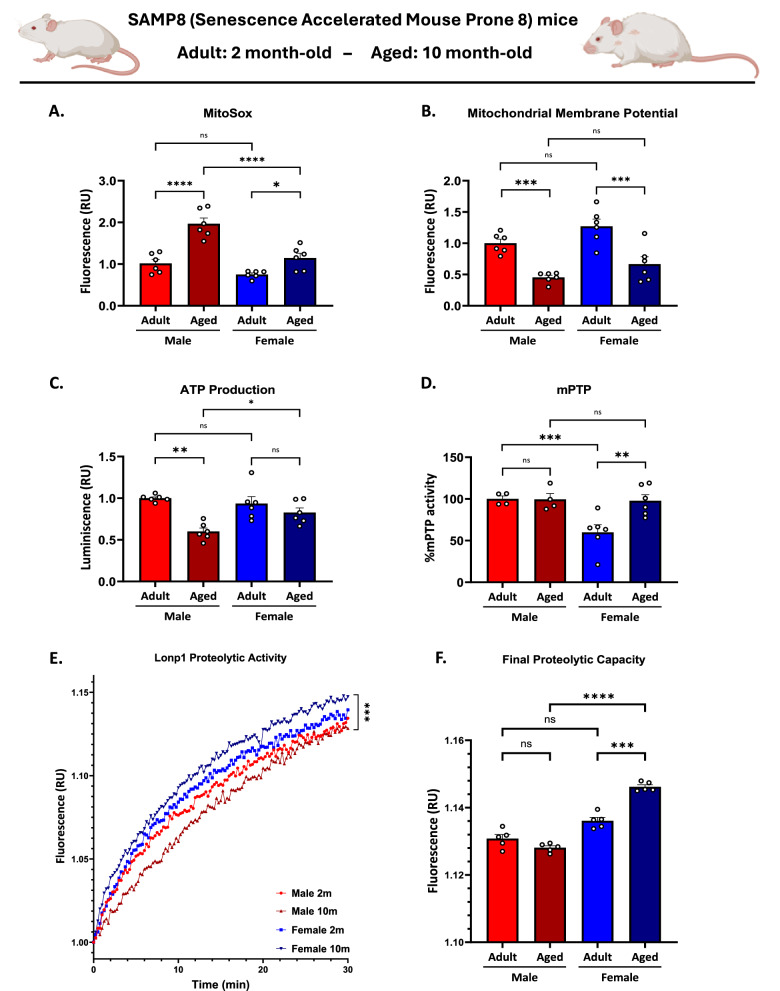
SAMP8 mice recapitulate sex-specific mitochondrial dysfunction observed in C57BL/6 J mice. Mitochondrial function was evaluated in male and female adult (2-month-old) and aged (10-month-old) SAMP8 mice. **A** Mitochondrial superoxide production of an isolated mitochondrial-enriched fraction, measured by the fluorescent dye MitoSox. **B** Mitochondrial membrane potential and (**C**) ATP production were measured in an enriched mitochondrial fraction exposed to oxidative substrates. **D** Measurement of mPTP activity in isolated mitochondria through a fluorescent mPTP assay. **E** Lonp1 proteolytic activity was evaluated using a FITC-casein degradation assay, with fluorescence measurements taken every 15 s up to 30 min. **F** Quantification of final proteolytic capacity derived from FITC-casein degradation curves. Values represent means ± SEM. Statistical differences were calculated by two-way ANOVA. *p < 0.05, **p < 0.01, ***p < 0.001, ****p < 0.0001.

The Senescence-Accelerated Mouse Prone 8 (SAMP8) strain is a non-transgenic murine model that exhibits an accelerated aging phenotype characterized by early cognitive decline, mitochondrial dysfunction, and increased oxidative stress [61, 62]. To assess whether the mitochondrial alterations previously observed in aged C57BL/6 J mice were also present in this accelerated-aging model, we analyzed 2 (adult) and 10-month-old (aged) male and female SAMP8 mice. Mitochondrial function was evaluated through multiple complementary parameters, considering those that support sex differences in aging in C57BL/6, including mitochondrial superoxide production (MitoSOX), mitochondrial membrane potential (MMP), ATP production, mitochondrial permeability transition pore (mPTP) activity, and Lonp1 proteolytic activity (Fig. 7). First, we analyzed mitochondrial superoxide production using a mitochondrial-enriched fraction of the hippocampus from SAMP8 mice. Surprisingly, the results were similar to those observed in C57BL/6 mice, where aged males generate higher levels of superoxide ions. Two-way ANOVA analysis revealed significant effects of sex [F_(1,20)_ = 27.98; *p* < 0.001] and age [F_(1,20)_ = 42.87;*p* < 0.001], as well as a significant sex × age interaction [F_(1,20)_ = 7.35; *p* = 0.01]. Post hoc analyses showed that aged males exhibited markedly higher mitochondrial superoxide production than both adult males and aged females, whereas aged females also displayed higher mitochondrial superoxide production than adult females (Fig. 7A). Then, we measured the mitochondrial membrane potential, statistical analyses revealed there were significant main effects of sex [F_(1,20)_ = 7.27; *p* = 0.01] and age [F_(1,20)_ = 41.18; *p* < 0.001], though the interaction was not significant (*p* = 0.75). Based on post hoc comparisons, these results indicate that adult males had higher mitochondrial membrane potential than aged males; similarly, the same pattern was observed in females (Fig. 7B). Finally, we measured mitochondrial ATP production to complete the mitochondrial bioenergetics analyses, and our results showed that mitochondrial energy generation also declined with age. Two-way ANOVA analysis revealed a significant effect of age [F_(1,20)_ = 21.37; *p* < 0.001] and a sex × age interaction [F_(1,20)_ = 7.10; *p* = 0.01], while no main effect of sex was detected (*p* = 0.15). Post hoc comparisons revealed that aged males exhibited a stronger reduction in mitochondrial ATP production compared to both adult males and aged females (Fig. 7C). Additionally, considering that aged female C57BL/6 mice present higher mPTP activity compared with males, we measured this parameter in SAMP8. Interestingly, this same pattern is observed in aged SAMP8 mice. For mPTP activity, we found significant main effects of sex [F_(1,12)_ = 26.00; *p* < 0.001] and age [F_(1,12)_ = 6.49; *p* = 0.02], as well as a significant sex × age interaction [F_(1,12)_ = 7.06; *p* = 0.02]. Post hoc analysis revealed that while adult males exhibited higher mPTP activity than adult females, aged females showed a marked increase compared to both adult females and aged males (Fig. 7D). Thus, these findings demonstrate that SAMP8 mice recapitulate the sex-dependent mitochondrial alterations observed in C57BL/6 J mice. Aged male SAMP8 mice exhibit exacerbated mitochondrial oxidative stress, decreased membrane potential, and impaired ATP production, whereas aged females show enhanced mPTP activity.

Finally, Lonp1 proteolytic activity was assessed by monitoring FITC-casein degradation assays. The fluorescence curve revealed slower substrate degradation in aged males, reflecting a substantial decline in Lonp1 proteolytic capacity (Fig. 7E). Quantification of the final proteolytic capacity confirmed that Lonp1 activity was significantly reduced in aged males compared to aged females. Statistical analyses reveal that there was a significant effect of sex [F_(1,96)_ = 41.38, *p* = 0.001], a significant effect of age [F_(1,96)_ = 4.14, *p* = 0.005], as well as a significant sex × age interaction [F_(1,96)_ = 12.37, *p* = 0.001]. Post hoc analyses revealed that 2-month-old females exhibited lower levels compared to 10-month-old females. Moreover, 10-month-old females showed higher values than males of the same age (Fig. 7F). Interestingly, in contrast to C57BL/6 mice, aged females SAMP8 showed increased Lonp1 activity, suggesting some compensatory mechanism to stimulate Lonp1 proteolysis in aging. This effect is not observed in aged males, which inclusive tend to decrease Lonp1 function.

Therefore, altogether, these results indicate that mitochondrial and proteostatic dysfunctions associated with aging are conserved across these strains, reinforcing the generalizability of the observed sex-dependent differences in hippocampal mitochondrial physiology.

## Discussion

Understanding the mechanisms underlying the aging process is of utmost importance, mainly if we focus on the sex differences that may appear during aging. Gaining such insights is essential for developing sex-dependent preventive and therapeutic strategies that can improve the quality of life in the aging population and mitigate the impact of age-related diseases. Overall, there is a notable lack of studies directly comparing the rate of aging between sexes under the same experiments. In this study, we studied whether sex influences age-related changes in spatial cognition and whether these effects are associated with mitochondrial dysfunction in a sex-dependent manner. We found that short-term memory is more impaired in aged males, while long-term memory shows no significant differences in both aged sexes. Interestingly, recognition memory tends to fail in aged females, whereas spatial localization memory is more impaired in aged males. In males, this cognitive decline correlates with higher oxidative stress, potentially evoked by higher superoxide production in mitochondria and reduced mitochondrial bioenergetics. In contrast, aged females exhibit greater mitochondrial permeability transition pore (mPTP) activity. Additionally, we observed that both general and mitochondrial proteostasis deteriorate in a sex-dependent manner during aging.

Sex-related differences in cognition have been extensively studied in adult mammals [63]. However, the knowledge concerning the involvement of aging in sex-dependent cognitive performance is scarce and somewhat conflicting. Spatial learning and memory decline with age [64]. However, memory outcomes observed in aged individuals depend not only on the age of the animals but also on the context of the behavioral task used for assessment. For example, when evaluating working memory, aged males show more pronounced memory impairment in the multi-branched maze test [65]. In other cases, memory impairment is seen in females using the Morris water maze test [66], and even non-sex-dependent impairment has been reported [67]. In our study, using the MWM test, we found that aged males exhibited more significant short-term memory impairment during the initial learning phase compared to aged females. However, long-term memory performance eventually reached similar levels between the two aged groups. Aged males learn more slowly than females, but learn the task in due course. Despite this, during the probe trial, aged males outperformed aged females, suggesting that although males learn the task more slowly, they retain spatial memory more precisely once learned.

Additionally, we found that object recognition was affected in a sex-dependent manner in aged animals. Aged males preferentially explored the novel object in the NOR test, whereas aged females exhibited no such preference, which agrees with findings in aged rats [68]. However, other studies report no sex-related differences in the NOR or NLR tests [66]. In contrast, our results revealed that aged females outperform males, as demonstrated by the lack of preference for the novel object location in male mice. It is worth noting that this pattern extends to other cognitive domains. For example, aged females outperform their male counterparts in social recognition tasks [69]. Collectively, these findings suggest that different cognitive functions are altered during aging in a sex-specific manner, highlighting the need for more exhaustive behavioral analysis.

Previous studies have shown that aged female rats outperformed aged males in a physical rotarod test measuring locomotor activity [67, 70]. However, other studies have observed no sex differences in locomotor activity during aging using the open-field test [66, 69]. Here, we observed similar distance traveled and speed in aged mice of both sexes in the open field arena. These results suggest that while forced or stress-inducing physical tests, such as the rotarod, may reveal sex-related differences in locomotor capacity, voluntary locomotor tests may not. Additionally, we found that aged mice of both sexes did not exhibit anxiety-like behavior. Some studies indicate that aged males show higher anxiety-like behavior than females [66], while others report that aged females, but not males, exhibit heightened anxiety [71]. These discrepancies might be attributed to methodological differences, such as variations in arena dimensions [72] or differences in husbandry conditions between facilities. [73–75].

Oxidative stress is a well-established hallmark of aging [76] and has been linked to hippocampal memory decline [13]. Our data showed that ROS levels, both in whole hippocampal lysate and in cytoplasmic and mitochondrial subcellular fractions, are elevated mainly in aged male mice. Mitochondrial superoxide production was also markedly increased in males. These sex-related differences in oxidative stress are consistent with prior findings that suggest males are more susceptible to oxidative damage, which has been associated with the development of neurodegenerative disease risk [77]. Previous studies have reported that adult males showed greater DNA damage and protein carbonylation due to increased peroxide production and reduced antioxidant capacity compared to females [17, 78]. To investigate the potential mechanisms underlying these sex differences, we examined the levels of antioxidant enzymes and found that SOD1 and GSR were elevated in the aged female hippocampus, consistent with our previous work [28]. In contrast, in males, these enzymes remained unchanged. Although Borras et al. reported higher antioxidant enzyme levels in adult females compared to males, we did not observe such differences in our adult group [17]. There is still controversy regarding the levels of antioxidant proteins in aged males associated with the Nrf2 pathway, such as SOD1 and GSR [79]. Therefore, further studies are needed to clarify whether the changes observed here are due to impaired Nrf2 signaling. Overall. Our results suggest that aged female mice may possess a more robust antioxidant response, potentially allowing them to better cope with oxidative stress during aging.

Due to the inherent characteristics of the male and female nature, we must consider the hormonal role in regulating oxidative stress [80]. It is reported that testosterone levels decrease in males during aging; however, the effects of this decline on antioxidant responses vary across studies, which may be tissue-specific [81]. In the case of females, although that is reported that female hormones like estrogen and progesterone have a positive regulation in the expression of antioxidant enzymes [81], in aging, the levels of sex hormones have been reported to be reduced in both sexes [76], thus damping the protective effects of hormones. Moreover, we observed that aged females have increased levels of antioxidant proteins, suggesting another mechanism that regulates these differences in antioxidant response [82, 83]. Despite this, pathological aging is shown with sex differences by sex chromosomes, with male mice more prone to mitochondrial failure in multiple sclerosis and Alzheimer’s disease models [84, 85], suggesting a common mechanism between physiological and pathological aging.

Mitochondria are essential structures in cellular bioenergetics and metabolism. In this way, these organelles are involved in aging and lifespan determination and may play a role in age-related neurodegenerative disorders [28, 86]. In line with previous reports from our group and others, diverse animal models have shown that mitochondrial function declines with age. Old mice display a diminished mitochondrial function compared to young mice, which contributes to the deterioration in the structural function of the organisms in an age-dependent manner [28, 36, 87–89]. Interestingly, these mitochondrial differences may also exhibit sex dependency, considering that sex hormones play a critical role in regulating energy metabolism [88–90]. Interestingly, several studies showed that the age-induced changes in sex steroid levels observed in specific tissues, such as the liver, muscle, and blood, may be different from those observed in the brain; this is because the brain pool of sex steroids depends on both endocrine gland production and the local synthesis of neurosteroids [90, 91].

Thus, a recent pilot study in healthy human adults (mean age 30.8 ± 7.1 years) detected sex-associated differences in mitochondrial function [91]. They evaluated mitochondrial bioenergetic parameters, including respiratory capacity, activity of mitochondrial respiratory complexes, and electron transport, in peripheral blood mononuclear cells (PBMCs) and the brain tissue. This study revealed that these factors in PBMCs isolated from female blood samples were significantly higher than in males. In this way, the ATP levels of female participants were approximately 10% higher than those of males. On the other hand, cerebral parameters such as N-acetylaspartate (NAA), a marker of neuronal energy consumption in the brain, were significantly higher in female than in male participants. This is interesting because the NAA concentration correlates with mitochondrial function, and low NAA levels may indicate mitochondrial dysfunction. Therefore, females exhibited significantly higher mitochondrial bioenergetic function in PBMCs and brains than males in adulthood. [91].

Furthermore, some studies have shown no differences between males and females of the B6 (C57Bl/6 J) mouse strain in heart, skeletal muscle, and liver in various mitochondrial bioenergetic parameters, including mitochondrial oxygen consumption and ATP content [88, 92]. However, in the brain, it was observed that young female brain mitochondria showed a significant increase in bioenergetic parameters, such as mitochondrial respiratory function and oxygen consumption, compared with males of the same age [92]. In contrast, we did not find sex differences in adult mice concerning mitochondrial membrane potential and ATP production. However, the total ATP content varied between aged mice in a sex-dependent manner. Although mitochondrial function does not show differences, variations in cytoplasmic ATP could be explained by differences in other factors, such as glycolysis. This metabolic pathway is influenced by a combination of enzymatic factors, substrates, hormonal regulation, and cellular conditions [93, 94].

Additionally, it appears that the mitochondrial effects of sex hormones are more pronounced in the brain than in other organs and tissues. Furthermore, another study in male and female C57BL/6 mice at two ages, 3-month-old and 20-month-old, showed that brain mitochondrial metabolism is sexually dimorphic. In young and aged mice, they evaluated mitochondrial oxygen consumption and the activities of mitochondrial complexes. They observed an elevated mitochondrial metabolism in young females compared to young males, and these characteristics were not observed in aged mice independently of the sex [90].

Mitochondria also regulate calcium homeostasis; a significant increase in mitochondrial Ca^2+^ uptake opens the mitochondrial permeability transition pore (mPTP), leading to mitochondrial swelling and apoptosis [28]. Several studies have reported that mitochondrial dysfunction in aging promotes the activation of mPTP [28, 36, 95]. Nevertheless, there is not much evidence that the activation of mPTP can be sex-dependent during aging. Thus, we are the first to describe that mPTP is differentially activated between sexes in an age-dependent manner.

These results offer an interesting perspective on the structure and molecular regulation of sex-specific variations in the mPTP during aging. In contrast to males, aged females showed increased levels of ANT and VDAC, proteins that mediate adenine nucleotide exchange and calcium flux across mitochondrial membranes, respectively [96, 97]. This could contribute to the improved mPTP activity in females, because these proteins have been proposed as regulatory proteins rather than structural elements of the mPTP [97, 98]; enhancing the predisposition to mPTP opening [97, 99, 100]. In addition, despite older males displaying elevated levels of CypD, aged females present significantly higher levels compared to aged males. CypD is a crucial regulator of mPTP opening, which makes mitochondria more susceptible to calcium overload. These different molecular characteristics suggest that the processes underlying mPTP activation in aging are sex-dependent, considering that aged females exhibit increased protein regulatory and structural mPTP proteins. In contrast, aged males seem to be dependent on CypD-mediated mPTP formation, but to a lesser extent than aged females. Interestingly, our findings showed no apparent age- or sex-dependent changes in proteins such as OSCP, an ATP synthase subunit that has been linked to pore formation in the past. These results are consistent with studies that question the direct presence of OSCP protein in the structural function of pore identity, and instead consider OSCP a protective agent. This is due to studies demonstrating that its overexpression protects against mPTP activation and neurodegeneration [98, 99, 101–103].

On another hand, pore-associated proteins, structure and activity of the mPTP may be altered by mitochondrial functions, such as mitochondrial membrane potential and mitochondrial calcium buffering. Several studies have demonstrated that aging is associated with impaired calcium regulation through the Na^+^/Ca^2+^ exchangers and the mitochondrial calcium uniporter, making the mitochondria more susceptible to calcium overload [104, 105]. Furthermore, variations in the calcium homeostasis of neurons between the sexes propose that female mitochondria may be more susceptible to Ca^2+^ overload-induced pore activation [106], which is consistent with our results. Finally, aging-induced reductions in mitochondrial membrane potential (ΔΨm) increase the probability of pore opening [95, 107]; therefore, this could also contribute to mPTP in our aged mice. Thus, these findings highlight the complex regulation of mPTP and support the idea that several molecular pathways may interact to promote mitochondrial pore opening as individuals age, sex-specifically. In this context, it is interesting to establish the principal contribution of these protein alterations to mPTP activity. Additional validation of this mechanism will be necessary, using pharmacological inhibitors and genetic approaches. Several assays in models of calcium overload, ischemia, and neurodegeneration have shown that sanglifehrin A and cyclosporin A have neuroprotective effects by binding to CypD and preventing mPTP opening [100, 108, 109]. In addition, CypD knockout mice (Ppif -/-) exhibit delayed neurodegeneration, protection against ischemic brain injury, and notable resistance to calcium-induced mPTP opening [109]. Thus, sex-dependent mPTP regulation during aging most likely entails both functional alterations in calcium handling and ΔΨm as well as differential remodeling of pore-associated proteins, which combine to form unique mitochondrial vulnerabilities in males and females.

The results related to mPTP, particularly in females, are also relevant because mPTP dysregulation has been increasingly implicated in neurodegenerative diseases, including Alzheimer’s disease (AD) [55], suggesting a direct link between mitochondrial aging and the predisposition to develop AD. Notably, women have a higher incidence of AD, and growing evidence indicates that sex-specific differences in mitochondrial function may contribute to this disparity. Our study demonstrates that aged females exhibit heightened mPTP activity, which could partly explain their higher predisposition to AD. Modulating mPTP activity could represent a promising approach to mitigating mitochondrial dysfunction and reducing AD risk, particularly in female individuals. Future research should focus on elucidating the molecular mechanisms underlying sex-specific mitochondrial vulnerabilities to improve prevention and treatment strategies for AD.

Maintaining proteostasis is crucial for cellular function and viability, especially in aging, where the balance between protein synthesis, folding, and degradation becomes increasingly challenging [110]. In the aging brain, the decline in proteostasis is associated with the accumulation of damaged proteins, leading to synaptic dysfunction and neuronal degeneration [111]. In this work, we observed an increase in the load of unfolded proteins in the hippocampus of aged males (Fig. 6), consistent with previous reports in the literature [29, 112]. Studies have shown that sex-related differences in proteostasis can impact tissue-specific sensitivity to protein aggregations and age-related diseases [113]. Hormonal changes, such as estrogen deprivation, have been associated with accelerated aging and increased susceptibility to age-related diseases [114]. It has also been shown that this hormone can mediate specific responses to misfolded proteins, emphasizing the importance of hormonal regulation in maintaining proteostasis and cellular health [114]. Further studies are needed to clarify whether the changes observed are specific to the tissue studied and to determine the causes of these changes.

Mitochondria play a central role in maintaining proteostasis, with mitochondrial chaperones and proteases being essential for proper protein folding and degradation within the organelle, particularly in aging, where the integrity of mitochondrial proteins becomes increasingly compromised [115]. By analyzing the unfolded protein content in the mitochondria-enriched fraction, a significant increase in unfolded protein load was observed in a sex-independent manner in aged animals. Despite this, we observed a substantial reduction of HSP60 in the aged male group compared to the aged female group.

Finally, evaluating Lonp1, OMA1, and ClpP proteases, we observed that Lonp1 decreased in both age groups, with a more significant decrease in aged males. Although the mechanisms regulating Lonp1 expression remain poorly understood, recent findings suggest a positive relationship between the mRNA methyltransferase METTL3 and Lonp1 expression in the mouse kidney. It is noteworthy that, during aging, METTL3 levels decrease, as does Lonp1 expression, and an overexpression of METTL3 restores Lonp1 protein levels as well [116]. Similarly, the implication of Sirtuin-3 has been studied in the promotion of the mitochondrial unfolded protein response. It shows that Sirtuin-3 can promote the expression of Lonp1, mediated by the expression of CHOP, HSP10, and HSP60 previously, as a chain effect [117]. However, these observations have not been evaluated in terms of sex, leaving open the possibility that this regulatory mechanism of Lonp1 expression may differ between males and females. Also, ClpP levels were lower in aged females compared to adult females; however, adult females exhibited higher ClpP levels than adult males. Additionally, we observe that the proteolytic activity of Lonp1 in the hippocampus is decreased in both aged groups; even the male group had lower basal activity compared to the female group; however, the reason for this decrease remains one of the main unknowns of this study. Although we observed that females present higher Lonp1 proteolytic activity at baseline, which would explain why they retain a higher proteolytic capacity during aging compared to males, it is essential to delve deeper into the molecular and cellular mechanisms that could be regulating Lonp1 proteolytic activity to understand the role of this protease in brain aging fully. Specifically, the decrease in the proteolytic activity of Lonp1 in aging raises new questions about the post-translational modifications that might occur in this protein. It has been described that increased ROS can generate post-translational modifications in Lonp1, which affect its proteolytic activity [118]. This finding is consistent with our results, which show that males produce more ROS than females during aging. However, it is also described that other modifications exist, such as the phosphorylation of Lonp1 by Akt kinase [119], which may be differential by sex. It has been shown that cultured adult cardiomyocytes from female mice exhibit greater Akt activation due to stimulation via the estrogen receptor [120], so this is probably one of the possible mechanisms by which females have higher basal Lonp1 activity. Additionally, has been described that Lonp1 can undergo Sirt3-regulated acetylation, which enhances its proteolytic activity, whereas deacetylation promotes Lonp1 degradation [121]; however, there is no evidence associated with Sirt3 dysregulation between sexes in physiological states. It could also differentially alter Lonp1 function in males and females, affecting its ability to perform its proteolytic function and thus contributing to the accumulation of abnormal proteins in the mitochondria. Future studies could validate these possibilities. Besides, this sex distinction highlights a marked male predisposition to mitochondrial proteostasis failure in aging. This sex difference raises the question of possible underlying mechanisms contributing to this observed disparity. In this context, studies have shown that the interplay between sex hormones and proteostasis extends to mitochondrial function, where estrogen has been demonstrated to influence mitochondrial proteostasis and quality control mechanisms [90]. It has been described that hormonal regulation can impact the expression and activity of components involved in mitochondrial proteostasis [122]. Thus, our results suggest that hormonal mechanisms may contribute to sexual dimorphism during aging.

Several studies report the impact of sex hormones on various cellular processes. This influence is determined by the availability of circulating hormones, which depends on the individual’s age, and by the presence of specific receptors, which are determined by the cell type. This suggests that circulating hormone levels are not necessarily a direct indicator of their effect. Many of our results show differences between females and males, which also suggests that it may be mediated, at least in part, by changes in sex hormone levels in aging. To resolve this inquiry, we measured the circulating hormonal levels in adult and old mice, and we found some variability among the data. To account for the high variability typical of hormone measurements, we analyzed age-related differences using geometric means (GM) and evaluated the fold-change. In males, testosterone levels were slightly lower in aged individuals compared to adult individuals, corresponding to a fold-change (Old/Adult) = 0.90, indicating an approximate 10% decline with age. In females, estradiol levels remained largely stable across age groups, with a fold-change = 0.97, suggesting only a ~ 3% decrease. Statistical comparisons revealed no significant differences between age groups for either testosterone or estradiol. Collectively, our results indicate that, within this sample analyzed, aging did not significantly alter basal circulating levels of testosterone in males or estradiol in females. However, a modest downward trend in testosterone with age may be present. When we measured the difference between the lowest and highest hormonal levels in mice, we found the greatest variability in the testosterone of adult males (2.9 times) compared to the old males (0.9 times). We observed a similar pattern with estradiol, where the highest variability was in adult female mice (6.9 times) compared to old females (3.0 times). These results suggest that aged mice (20 months old) have lower but also less variable hormonal levels compared to adult mice (3 months old). However, this is not necessarily an indication of lesser hormonal influence in aged animals.

In other mammals (e.g., rats and non-human primates), the natural or artificial depletion of hormones, such as during menopause, estropause, or after an ovariectomy, triggers an increase in plasma luteinizing hormone (LH) or follicle-stimulating hormone (FSH). This is due to the loss of negative feedback from the ovaries, which typically regulates pituitary hormone secretion [123, 124]. Similarly, as ovarian and testicular cells age and release fewer hormones, the pituitary gland compensates by increasing the production of these factors (positive feedback). However, a different response is seen in C57BL6 mice. Studies have shown that ovariectomized C57BL6 mice do not exhibit different levels of LH or FSH compared to control animals, indicating that the circulating concentration of hormones, such as estrogen, even in older animals, is sufficient to impede pituitary secretion [125, 126]. These findings suggest that hormonal fluctuations related to the cycle and aging in mice may be less determinant, and that more specific effects could be occurring at the cellular level, as we demonstrated. Our data confirm that older mice have measurable hormonal levels of both testosterone and estradiol. Thus, the differences we observed are independent of overall hormone levels but instead are influenced by cell type and sex.

Also, the observed changes could be attributed to a differential activation state between males and females of the mitochondrial unfolded protein response (mtUPR). mtUPR is a crucial stress response pathway that alleviates mitochondrial stress caused by mistranslated and unfolded proteins, promoting biogenesis and maintaining mitochondrial function [127]. This response involves the upregulation of mitochondrial proteases and chaperones to ensure proper protein folding and degradation within the mitochondria [128]. Studies have shown that estrogen receptors, such as ERα, regulate the mtUPR, suggesting a potential sex-specific modulation of proteostasis mechanisms [129]. However, our results should be considered a landscape for the state of mitochondrial proteostasis, rather than the mtUPR activation state.

To further assess the generalizability of our findings, we extended our analysis to the Senescence-Accelerated Mouse Prone 8 (SAMP8) strain, a non-transgenic model that exhibits spontaneous, early-onset cognitive and mitochondrial decline [61, 62]. Consistent with the results obtained in C57BL/6 J mice, SAMP8 animals also displayed a clear sex-dependent pattern of mitochondrial alterations during aging. Aged male SAMP8 mice showed exacerbated mitochondrial superoxide production, a pronounced decrease in mitochondrial membrane potential, and ATP generation. In contrast, aged females exhibited increased mitochondrial permeability transition pore (mPTP) activity, suggesting a higher susceptibility to calcium-induced permeability and mitochondrial stress. Interestingly, Lonp1-dependent proteostasis is also different between sexes during aging. Whereas aged male SAMP8 mice showed a subtle tendency to reduce Lonp1 proteolytic capacity, aged female SAMP8 mice exhibited a significant increase in their Lonp1 proteolytic activity, revealing a drastic difference between females and males in aging, which could lead to aged females being more protected against mitochondrial proteolytic stress. Importantly, these effects occurred despite the accelerated aging background of the SAMP8 strain, reinforcing that the sex-specific mitochondrial differences observed are not strain-restricted but reflect a conserved biological phenomenon. Together, these results strengthen the external validity of our findings and suggest that sex-dependent mitochondrial vulnerability is a robust feature of aging across genetic backgrounds, potentially explaining why males and females exhibit differential susceptibility to aging and neurodegenerative disorders.

Our findings highlight significant sex-dependent differences in cognitive aging, mitochondrial function, and proteostasis, underscoring the complexity of aging-related processes. The distinct impairments observed in memory performance between males and females suggest that aging does not affect cognitive domains uniformly across sexes. This may be explained by differential mitochondrial bioenergetics, oxidative stress responses, and proteostatic mechanisms, among other cell processes. Notably, the marked decline in mitochondrial proteostasis in aged males, primarily due to decreased Lonp1 activity, suggests a potential vulnerability to neurodegeneration, which may be exacerbated by oxidative stress-induced post-translational modifications. These insights emphasize the necessity of incorporating sex as a biological variable in aging research to refine therapeutic strategies tailored to mitigate cognitive decline and promote healthy aging. Further studies are required to elucidate the molecular pathways underlying these sex-specific aging differences and their implications for age-related neurodegenerative diseases.

## Conclusions and future directions

Understanding the mechanisms underlying sex differences in aging is crucial for developing strategies to improve quality of life and counteract age-related diseases. This study investigated cognitive decline and mitochondrial dysfunction in aged male and female C57BL/6 J mice to better understand sex-specific aging processes in the hippocampus. We found that aged males exhibited impaired spatial memory and increased oxidative stress, marked by elevated mitochondrial superoxide production and proteostasis failure. In contrast, aged females showed deficits in recognition memory and heightened mitochondrial permeability transition pore (mPTP) activity, suggesting increased mitochondrial vulnerability. Moreover, the inclusion of Senescence-Accelerated Mouse Prone 8 (SAMP8) mice confirmed that these sex-dependent mitochondrial alterations are conserved across strains, reinforcing their biological relevance beyond a single genetic background and reflecting a conserved biological phenomenon. This study provides a comprehensive analysis of cognitive and mitochondrial changes in aging, emphasizing the importance of considering sex as a key factor in aging research, showing, for the first time in the same study, a comprehensive analysis of cognitive and mitochondrial changes between sexes in the aged hippocampus.

## Supplementary information

Supplementary Figure 1

Original Western Blots

Supplementary information

## Data Availability

The datasets generated during and/or analyzed during the current study are available from the corresponding author upon reasonable request.
