# Supplementary figures and images for: Unraveling sex differences in age-related hippocampal decline: differential mitochondrial dysfunction, Lonp1-dependent mitochondrial proteostasis and mtROS production in aged C57BL/6 mice
Source: Cell Death Dis. 2025 Dec 30;17(1):155. doi: 10.1038/s41419-025-08360-y (PMC12858937; doi:10.1038/s41419-025-08360-y)

## Slide 1
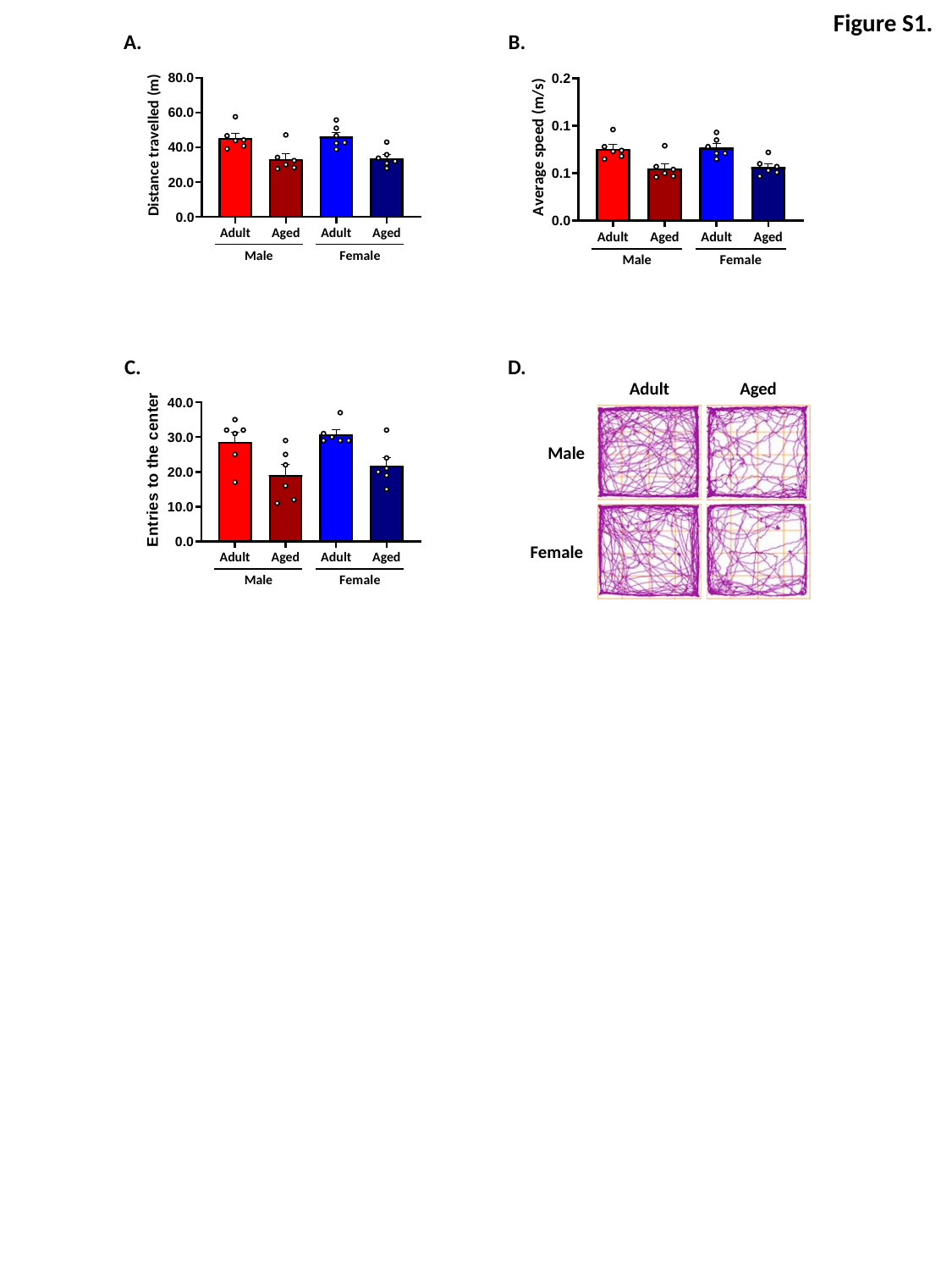

Figure S1.
A.
B.
C.
D.
Adult
Aged
Male
Female

Supplement: Supplementary file 1 — Supplementary Figure 1 [file 41419_2025_8360_MOESM1_ESM.pptx]
