# Supplementary material for: Unraveling sex differences in age-related hippocampal decline: differential mitochondrial dysfunction, Lonp1-dependent mitochondrial proteostasis and mtROS production in aged C57BL/6 mice
Source: Cell Death Dis. 2025 Dec 30;17(1):155. doi: 10.1038/s41419-025-08360-y (PMC12858937; doi:10.1038/s41419-025-08360-y)
Supplement: Supplementary file 3 — Supplementary information [file 41419_2025_8360_MOESM3_ESM.docx]

**Figure S1. Aged mice show reduced motility and increased anxiety independently of sex.** Behavioral performance was evaluated using the open-field test. (A) Total distance traveled (m). (B) Average velocity (m/s). (C) Number of entries to the center of the open field. (D) Graphical representation of the trajectories traversed by mice in the open field test. Values represent means ± SEM. Statistical differences were calculated by two-way ANOVA.
